# Supplementary material for: Constructing ordered and tunable extrinsic porosity in covalent organic frameworks via water-mediated soft-template strategy
Source: Nat Commun. 2024 May 8;15:3896. doi: 10.1038/s41467-024-48160-0 (PMC11079003; doi:10.1038/s41467-024-48160-0)
Supplement: Supplementary file 1 — Supplementary Information [file 41467_2024_48160_MOESM1_ESM.pdf]

Supplementary Information

Constructing Ordered and Tunable Extrinsic Porosity in Covalent Organic Frameworks *via* Water-Mediated Soft-Template Strategy

Ningning He,[1] Yingdi Zou,[1] Cheng Chen,[1] Minghao Tan,[1] Yingdan Zhang,[1] Xiaofeng Li,[2] Zhimin Jia,[1] Jie Zhang,[1] Honghan Long,[1] Haiyue Peng,[3] Kaifu Yu,[1] Bo Jiang,[1] Ziqian Han,[1] Ning Liu,[3] Yang Li,[1*] Lijian Ma[1*]

[1] *College of Chemistry, Key Laboratory of Radiation Physics & Technology, Ministry of Education, Sichuan University, Chengdu 610064, P.R. China*

[2] *Institute of Materials, China Academy of Engineering Physics, Mianyang 621907, P.R. China*

[3] *Institute of Nuclear Science and Technology, Key Laboratory of Radiation Physics and Technology of the Ministry of Education, Sichuan University, Chengdu 610064, P. R. China*

[*] *Corresponding authors: E-mail: ly7701850@163.com (Y. Li), ma.lj@hotmail.com (L. Ma)*

Table of Contents

Supplementary Section 1. Synthetic Methods 4

Supplementary Section 2. Adsorption Experiments 6

Supplementary Section 3. Screening and Optimization of Material Synthesis Conditions 7

Supplementary Section 4. TEM Images of DTAB/OTAB Self-Assembling into Micelles 9

Supplementary Section 5. DLS Data of DTAB/OTAB Self-Assembling into Micelles 12

Supplementary Section 6. XPS Spectra of MPCOF and OHMMCOF-DTAB/OHMMCOF-OTAB 13

Supplementary Section 7. BET Surface Area Plots and Pore Volume Parameters of MPCOF and OHMMCOF-DTAB/OHMMCOF-OTAB 14

Supplementary Section 8. EA of MPCOF and OHMMCOF-DTAB/OHMMCOF-OTAB 16

Supplementary Section 9. Water Contact Angles of MPCOF and OHMMCOF-DTAB/OHMMCOF-OTAB 17

Supplementary Section 10. TGA curves of MPCOF and OHMMCOF-DTAB/OHMMCOF-OTAB 18

Supplementary Section 11. SEM Images of MPCOF and OHMMCOF-DTAB/OHMMCOF-OTAB 19

Supplementary Section 12. TEM Images of MPCOF and OHMMCOF-DTAB/OHMMCOF-OTAB 20

Supplementary Section 13. EDS Mappings of MPCOF and OHMMCOF-DTAB/OHMMCOF-OTAB 21

Supplementary Section 14. Optimization of Experimental Conditions for Template Removal 23

Supplementary Section 15. XPS Spectra of OHMMCOF-1/OHMMCOF-2 26

Supplementary Section 16. BET Surface Area Plots and Pore Volume Parameters of OHMMCOF-1/OHMMCOF-2 27

Supplementary Section 17. EA of OHMMCOF-1/OHMMCOF-2 28

Supplementary Section 18. Water Contact Angles of OHMMCOF-1/OHMMCOF-2 29

Supplementary Section 19. TGA curves of OHMMCOF-1/OHMMCOF-2 30

Supplementary Section 20. SEM Images of OHMMCOF-1/OHMMCOF-2 31

Supplementary Section 21. TEM Images of OHMMCOF-1/OHMMCOF-2 32

Supplementary Section 22. EDS Mappings of OHMMCOF-1/OHMMCOF-2 33

Supplementary Section 23. HRTEM Images of OHMMCOF-1/OHMMCOF-2 34

Supplementary Section 24. Influence of Template Concentration on Experimental Results.. 36
Supplementary Section 25. BET Surface Area Plots and Pore Volume Parameters of OHMMCOF-1-0.5/3.0 eq 37
Supplementary Section 26. BET Surface Area Plots and Pore Volume Parameters of OHMMCOF-M-14:1/1:2 38
Supplementary Section 27. BET Surface Area Plots of OHMMCOF-3 39
Supplementary Section 28. Stability of MPCOF and OHMMCOF-1/OHMMCOF-2 40
Supplementary Section 29. Kinetic Model of MPCOF and OHMMCOF-1/OHMMCOF-2 .. 42
Supplementary Section 30. The Species Distributions of U(VI) and Th(IV) under Various Acidic Conditions 49
Supplementary Section 31. Experimental Results of MPCOF-Organic Synthesized by Organic Solvothermal Method 50
Supplementary Section 32. Time-Dependent Kinetic Adsorption Experiments and Kinetic Model of OHMMCOF-1-U(VI)/Th(IV) with Different Template Concentrations 52
Supplementary Section 33. XPS Spectra of OHMMCOF-2-U(VI)/Th(IV) 57
Supplementary Section 34. SEM and TEM Images and EDS Mappings of OHMMCOF-2-U(VI)/Th(IV) 59
Supplementary Section 35. Selective Adsorption Experiment of OHMMCOF-2 61
Supplementary Section 36. Comparison of Th(IV) Selective Adsorption Properties of OHMMCOF-2 and Other Porous Materials 62
Supplementary Section 37. Fractional Atomic Coordinates for the Unit Cell 63
Supplementary Section 38. Supplementary References 65

Supplementary Methods

Supplementary Section 1. Synthetic Methods

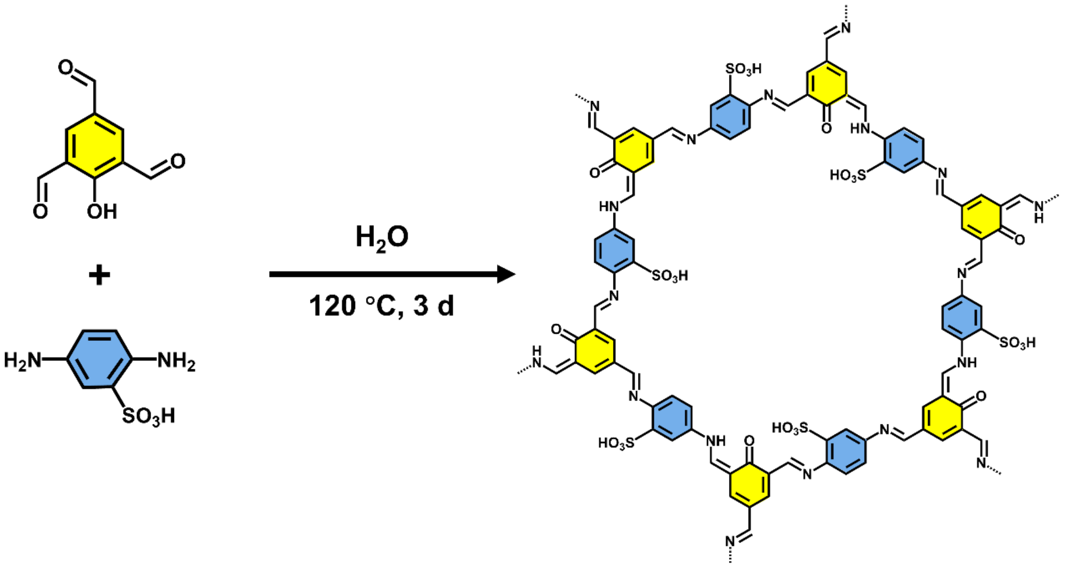

Supplementary Fig. 1 Synthesis of MPCOF.

S2.1 Synthesis of Triaminoguanidinium Chloride (TGCl)

Triaminoguanidine chloride (**TGCl**) was synthesized according to the previous reports.[1] Specifically, 1.91 g of guanidine hydrochloride was added to 10 mL of 1,4-dioxane followed by 3.41 g of hydrazine hydrate under agitation. Reflux the mixture for 3 h and then cool to room temperature. After filtration, the product was washed with 1,4-dioxane to remove excess hydrazine hydrate, and finally vacuum dried to obtain **TGCl** (yield: 95%).

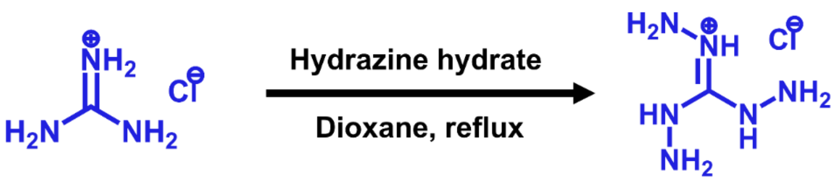

Supplementary Fig. 2 Synthesis of **TGCl**.

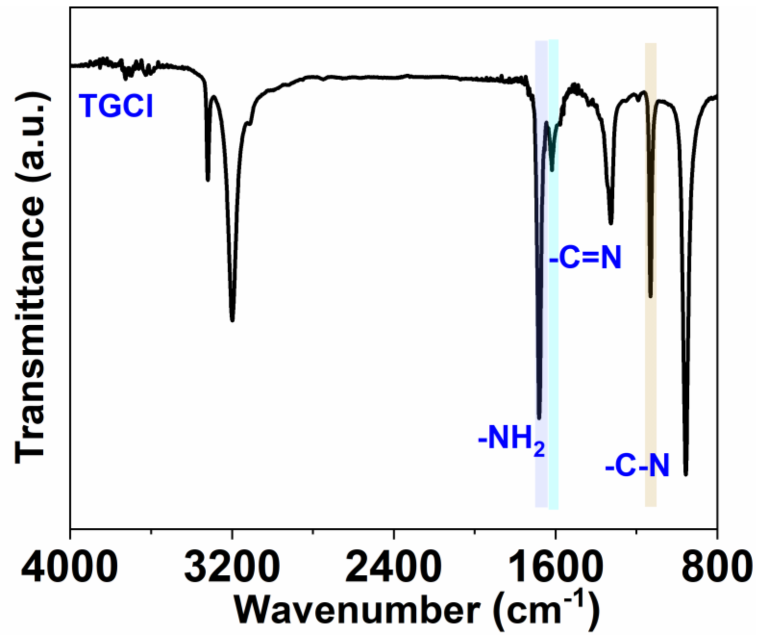

Supplementary Fig. 3 FT-IR spectra of **TGCl.**

Supplementary Section 2. Adsorption Experiments

All adsorption experiments were performed using a thermostatic shaking chamber at 25 °C with a vibration frequency of 200 rpm. The experiments were repeated more than three times and then averaged to ensure the reproducibility of the experimental results. Metal ion concentrations in solutions before and after sorption were determined using an ICP-OES OPTIMA8000 spectrometer. The equilibrium adsorption capacity (Q_e, mg/g), distribution coefficient (K_d, mL/g), and separation factor (*SF*) were calculated using the following equations:

$$Q_e = \frac{(C_0 - C_e)}{m} \times V \quad (1)$$

$$K_d = \frac{(C_0 - C_e)}{C_e} \times \frac{V}{m} \quad (2)$$

$$SF_{Th/M} = \frac{K_{d,Th}}{K_{d,M}} \quad (3)$$

where C_0 (ppm) and C_e (ppm) are the initial and equilibrium concentrations, respectively; V (L) is the volume of the solution; and m (g) is the mass of the adsorbent.

Supplementary Discussion

Supplementary Section 3. Screening and Optimization of Material Synthesis Conditions

To obtain COFs with ordered hierarchical micropores/mesopores, smaller 2-hydroxy-1,3,5-benzenetricarbaldehyde (Sa) building block with C_3 symmetry was selected to react with 2,5-diaminobenzenesulfonic acid (DABA) to construct micropores COFs. Firstly, by changing the type of solvents, it was found that only when H_2O was used as the solvent, the ordered arrangement peak of template self-assembly peaks would appear in the XRD pattern of the final synthesized COFs (**Supplementary Fig. 4a**). Subsequently, the crystallinity of the template self-assembly was further improved by changing the concentration of the catalyst (**Supplementary Fig. 4b**).

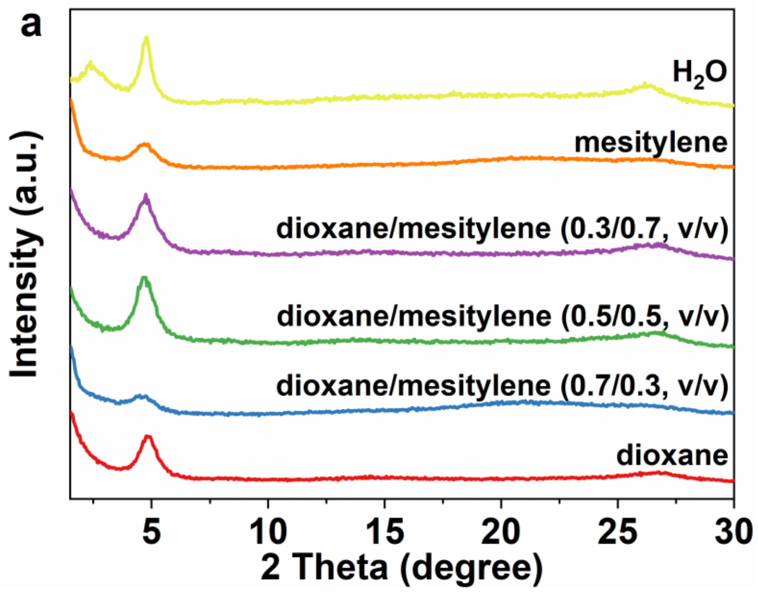

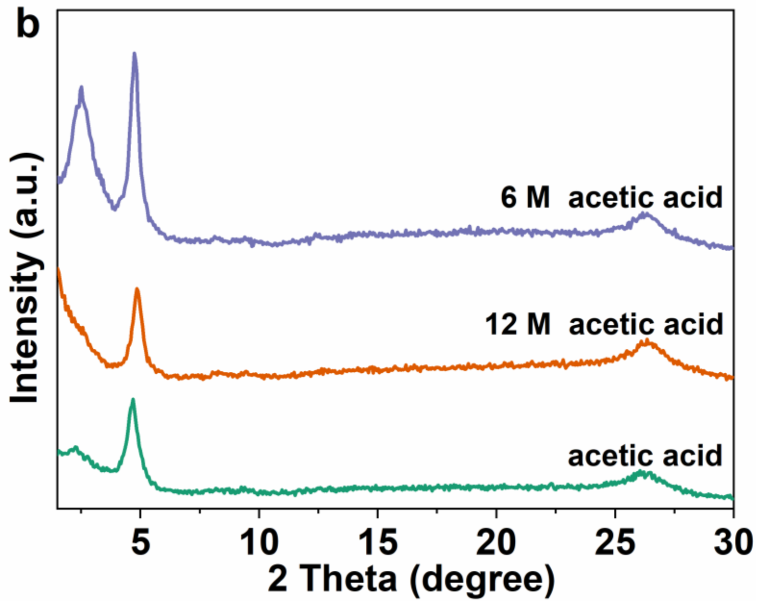

Supplementary Fig. 4 PXRD patterns of a) solvent screening and b) catalyst screening.

Supplementary Section 4. TEM Images of DTAB/OTAB Self-Assembling into Micelles

To gain further insights into the micelles state within our system, we first tried to directly observe the morphology of the micelles by TEM at room temperature, and the results are shown in **Supplementary Fig. 5**. According to **Supplementary Fig. 5**, we can see that both DTAB/OTAB exhibit spherical micelles and the Gaussian statistics can be used to obtain their average sizes of 3.29 and 4.45 nm, respectively, which is very close to the observed d-spacing calculated from the surfactant sizes and XRD patterns (3.4 and 4.3 nm, respectively). The larger micelles in the figure may be due to the agglomeration of micelles during TEM sample drying (**Supplementary Fig. 5a**). Subsequently, to our experimental conditions more closely, we also investigated whether the addition of acetic acid affected the morphology of the micelles (**Supplementary Fig. 6**). The results showed that the addition of acetic acid did not affect the micelle morphology of OTAB and DTAB.

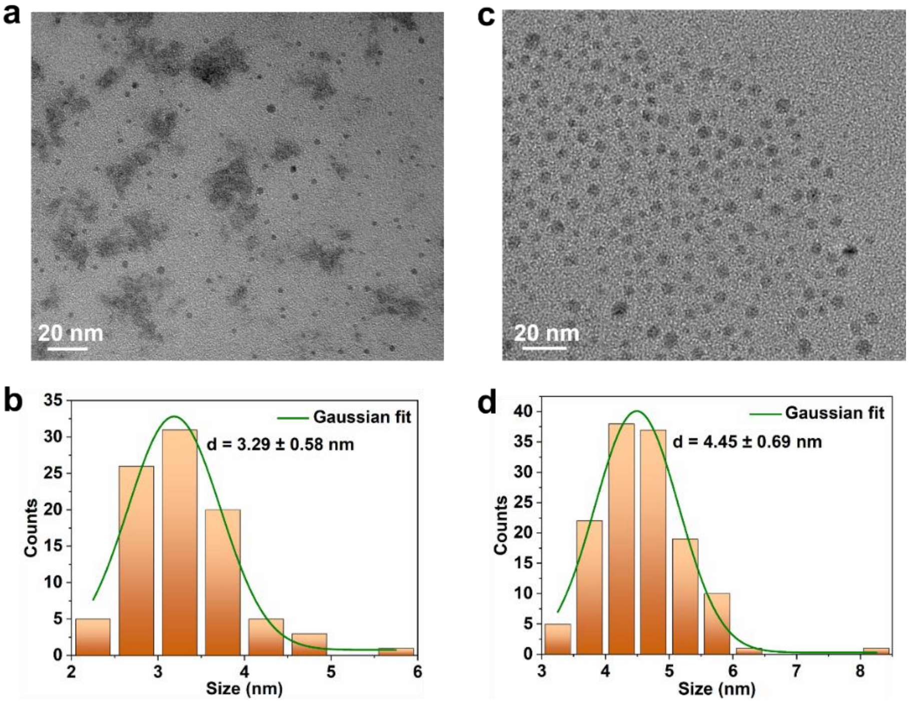

Supplementary Fig. 5 Micellar morphology observed by TEM at room temperature: a) DTAB and c) OTAB, and corresponding size distributions of spherical micelles: b) DTAB and d) OTAB.

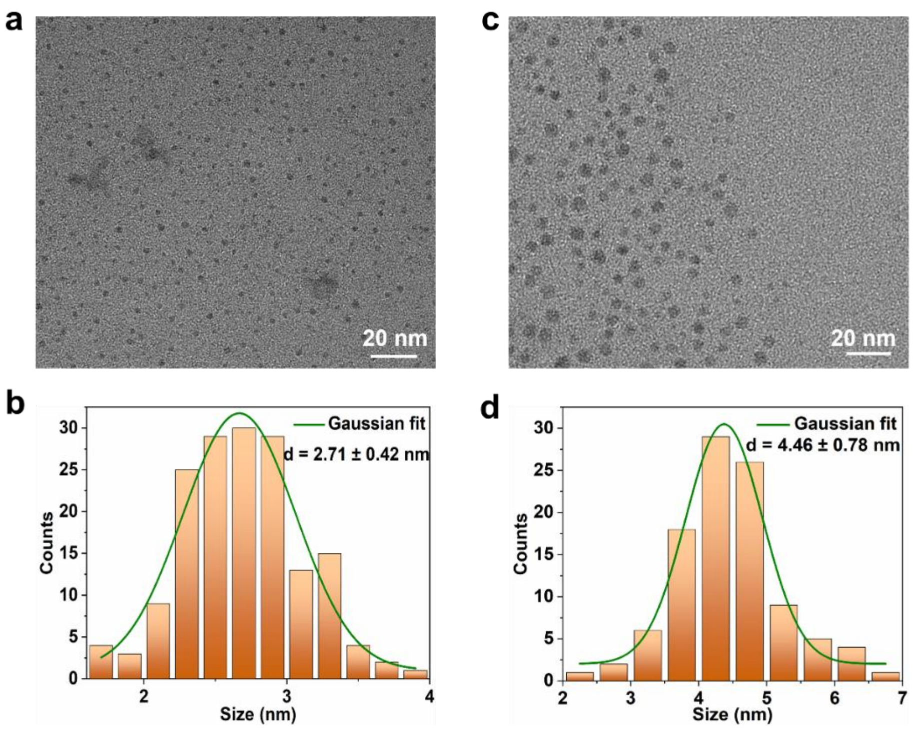

Supplementary Fig. 6 Micellar morphology observed by TEM with the addition of acetic acid at room temperature: a) DTAB and c) OTAB, and corresponding size distributions of spherical micelles: b) DTAB and d) OTAB.

To further approximate the synthesis conditions, we raised the temperature of the micelle assembly to 120 °C for 3 days in a sealed tube, after which the assembly product was collected for TEM observation, and the results are shown in **Supplementary Fig. 7**. When the temperature increases, both DTAB and OTAB exhibit a small number of spherical shapes accompanied by a large number of cylindrical micelle assemblies. The transverse dimensions of these cylindrical micelles are consistent with the DTAB/OTAB spherical micelles, approaching 3.4 and 4.3 nm, respectively, suggesting that they are derived from the further assembly of spherical micelles. Therefore, we conclude that the increase in temperature will lead to the further transformation of spherical micelles into cylindrical micelles. Similar examples of block copolymer assembly to form cylindrical micelles have been reported in the previous literatures.[2-4]

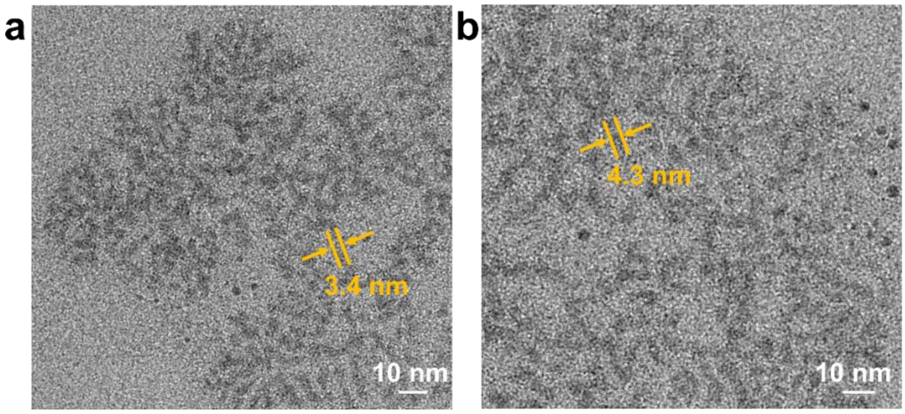

Supplementary Fig. 7 Micellar morphology observed by TEM after heating assembly in a 120 °C water bath for 3 d: a) DTAB and b) OTAB.

Supplementary Section 5. DLS Data of DTAB/OTAB Self-Assembling into Micelles

To further clarify the size of the micelles formed by the self-assembly of DTAB/OTAB, we also observed them using DLS testing, and the results showed that the particle sizes were distributed at 3.62/4.85 nm at 25 °C, respectively (**Supplementary Fig. 8**). The main reason for this difference in particle size is that the DLS test yields a hydraulic diameter, which is larger than the actual size. In addition, many larger-sized micelles resulting from disordered agglomeration were also observed. Finally, to investigate whether the increase in temperature will affect the morphology of micelles, we looked at whether the increase in temperature would affect the size of the micelles. The results showed that the addition of acetic acid did not affect the micelle morphology of OTAB and DTAB.

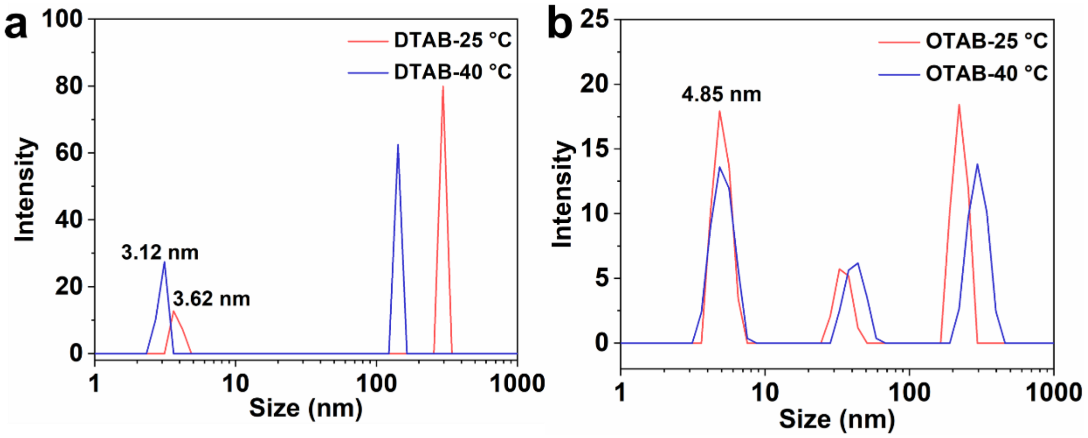

Supplementary Fig. 8 a) DTAB and b) OTAB micelles size distributions by DLS at different temperatures.

Supplementary Section 6. XPS Spectra of MPCOF and OHMMCOF-DTAB/OHMMCOF-OTAB

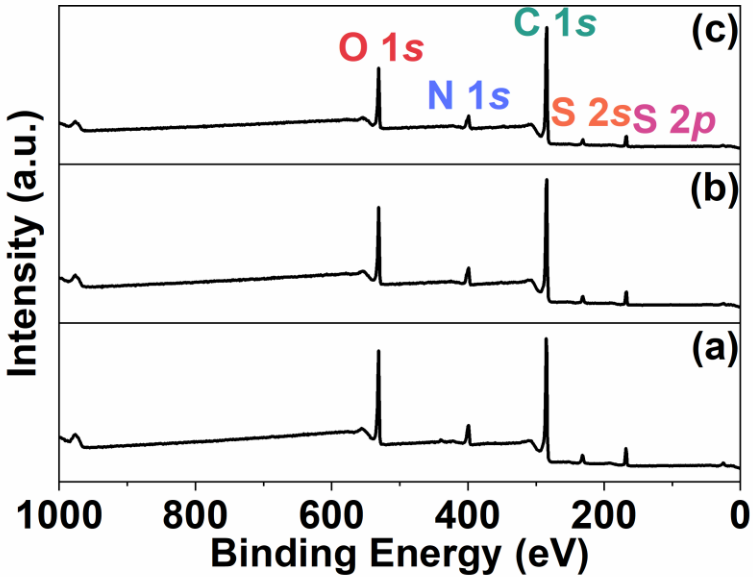

Supplementary Fig. 9 Typical XPS survey spectra of a) MPCOF, b) OHMMCOF-DTAB, and c) OHMMCOF-OTAB.

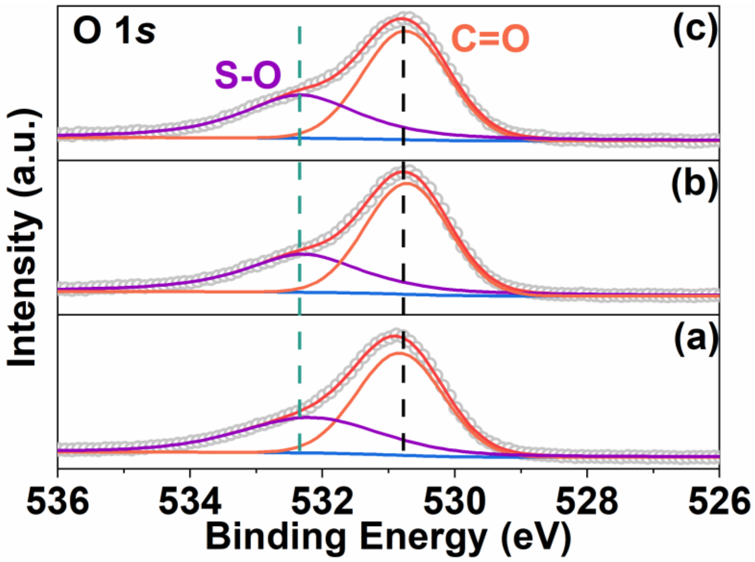

Supplementary Fig. 10 High-resolution XPS spectra of O 1*s* of a) MPCOF, b) OHMMCOF-DTAB, and c) OHMMCOF-OTAB.

Supplementary Section 7. BET Surface Area Plots and Pore Volume Parameters of MPCOF and OHMMCOF-DTAB/OHMMCOF-OTAB

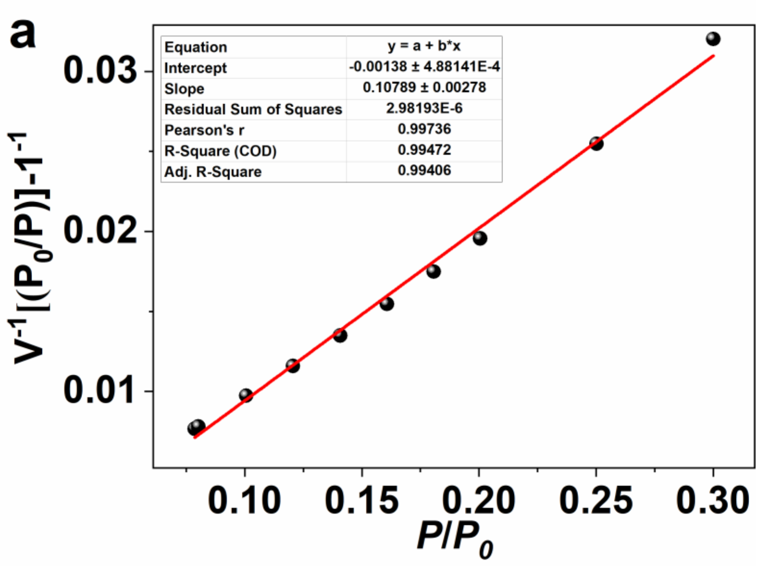

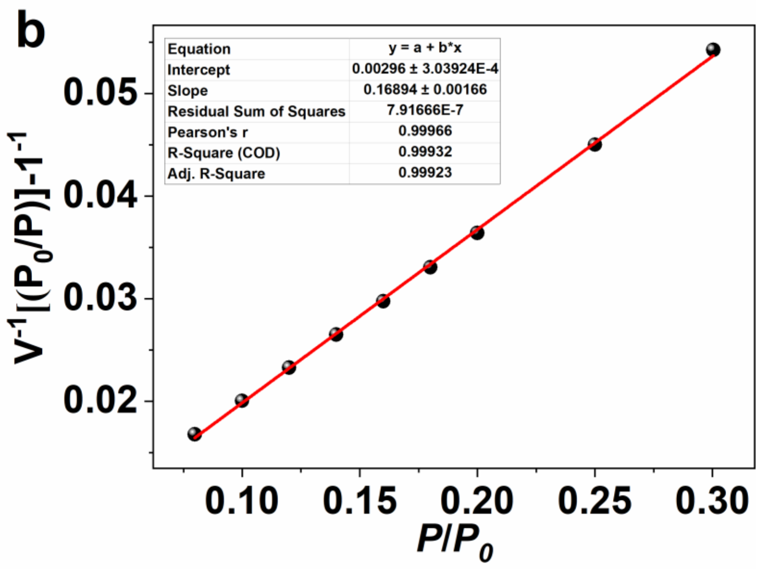

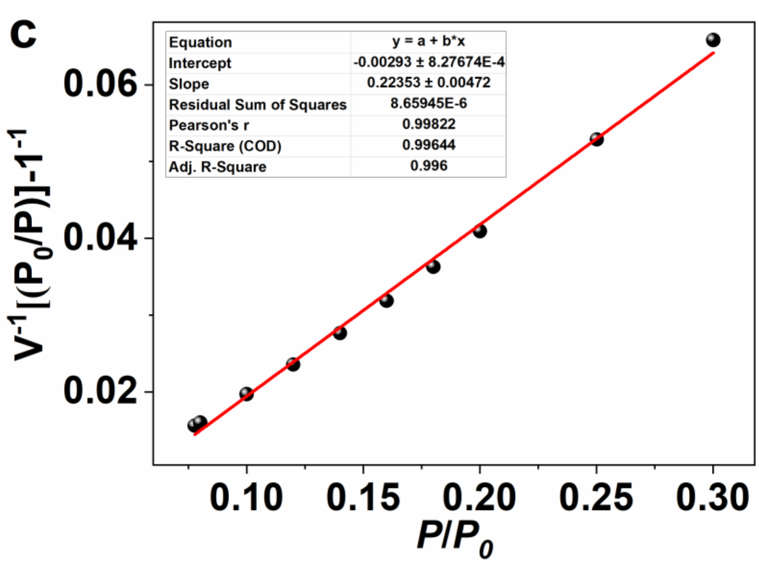

Supplementary Fig. 11 BET surface area plots of a) MPCOF, b) OHMMCOF-DTAB, and c) OHMMCOF-OTAB.

Supplementary Table 1. Pore volume parameters of MPCOF and OHMMCOF-DTAB/OHMMCOF-OTAB.

COF	V_{total} (cm^3/g)	$S_{BET\text{-}micro}$ (m^2/g)	$S_{BET\text{-}meso}$ (m^2/g)	$S_{BET\text{-}meso}$/ $S_{BET\text{-}micro}$
MPCOF	0.31	19.3	26.9	1.4
OHMMCOF-DTAB	0.17	15.6	20.7	1.3
OHMMCOF-OTAB	0.13	12.0	18.1	1.5

Supplementary Section 8. EA of MPCOF and OHMMCOF-DTAB/OHMMCOF-OTAB

Supplementary Table 2. EA results of MPCOF and OHMMCOF-DTAB/OHMMCOF-OTAB (Addition of water in molecular formula may originate in adsorbed water, or in unreacted functionalities at defect sites).[5]

COF	Chemical Formula		C/ wt%	H/ wt%	N/ wt%	O/ wt%	S/ wt%
MPCOF	$C_{36}H_{30}N_6O_{11}S_3\cdot(H_2O)_6$	Theoretical values	46.65	4.57	9.07	29.34	10.38
		Experimental values	46.76	4.58	8.83	29.97	9.86
OHMMCOF-DTAB	-	Experimental values	54.14	6.24	7.97	24.71	6.94
OHMMCOF-OTAB	-	Experimental values	54.03	6.34	7.47	26.77	5.39

Supplementary Section 9. Water Contact Angles of MPCOF and OHMMCOF-DTAB/OHMMCOF-OTAB

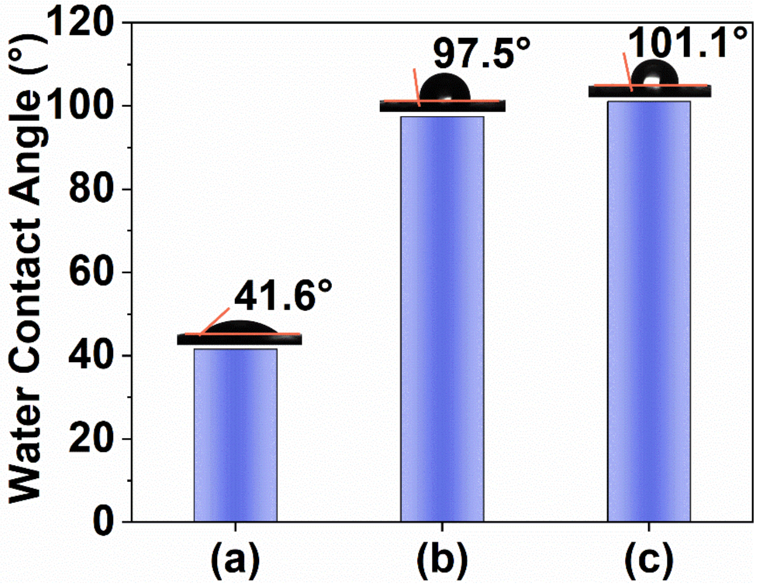

Supplementary Fig. 12 Water contact angles of a) MPCOF, b) OHMMCOF-DTAB, and c) OHMMCOF-OTAB.

Supplementary Section 10. TGA curves of MPCOF and OHMMCOF-DTAB/OHMMCOF-OTAB

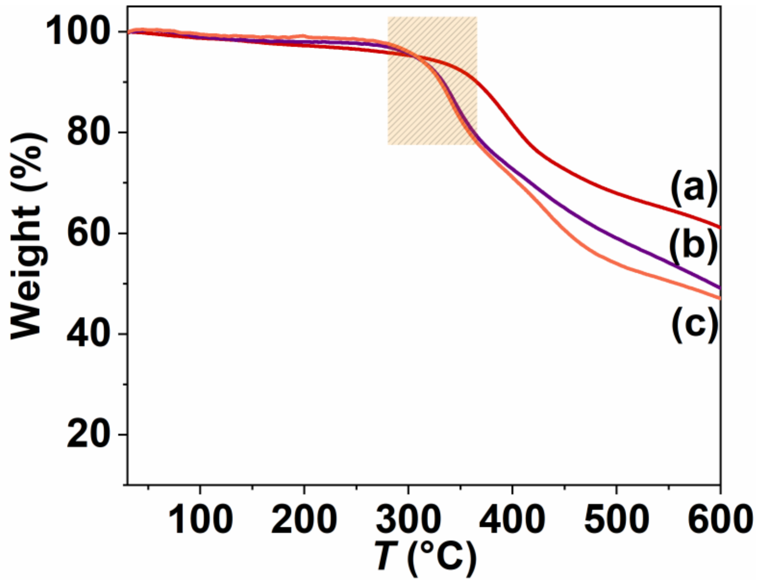

Supplementary Fig. 13 TGA curves of a) MPCOF, b) OHMMCOF-DTAB, and c) OHMMCOF-OTAB.

Supplementary Section 11. SEM Images of MPCOF and OHMMCOF-DTAB/OHMMCOF-OTAB

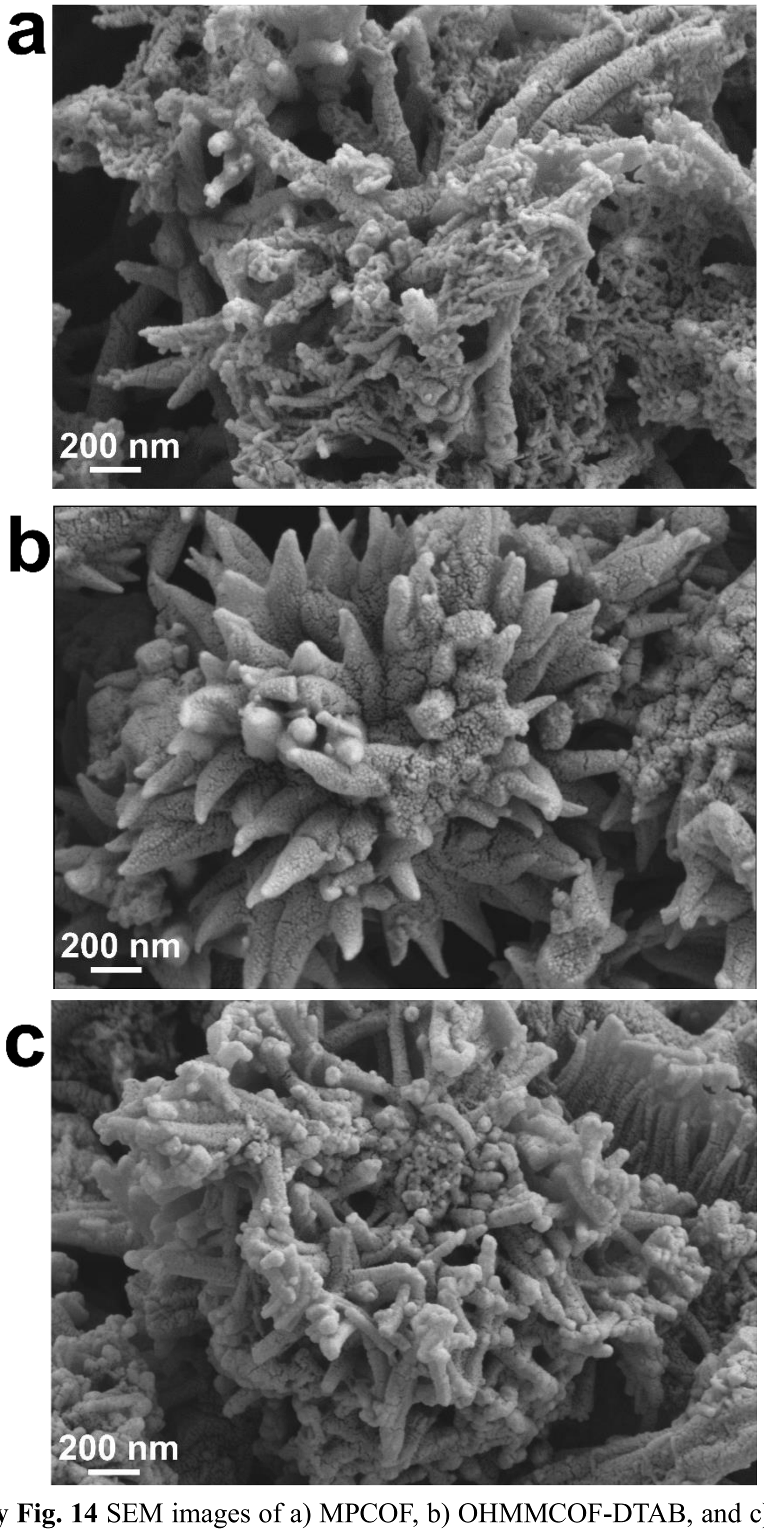

Supplementary Fig. 14 SEM images of a) MPCOF, b) OHMMCOF-DTAB, and c) OHMMCOF-OTAB.

Supplementary Section 12. TEM Images of MPCOF and OHMMCOF-DTAB/OHMMCOF-OTAB

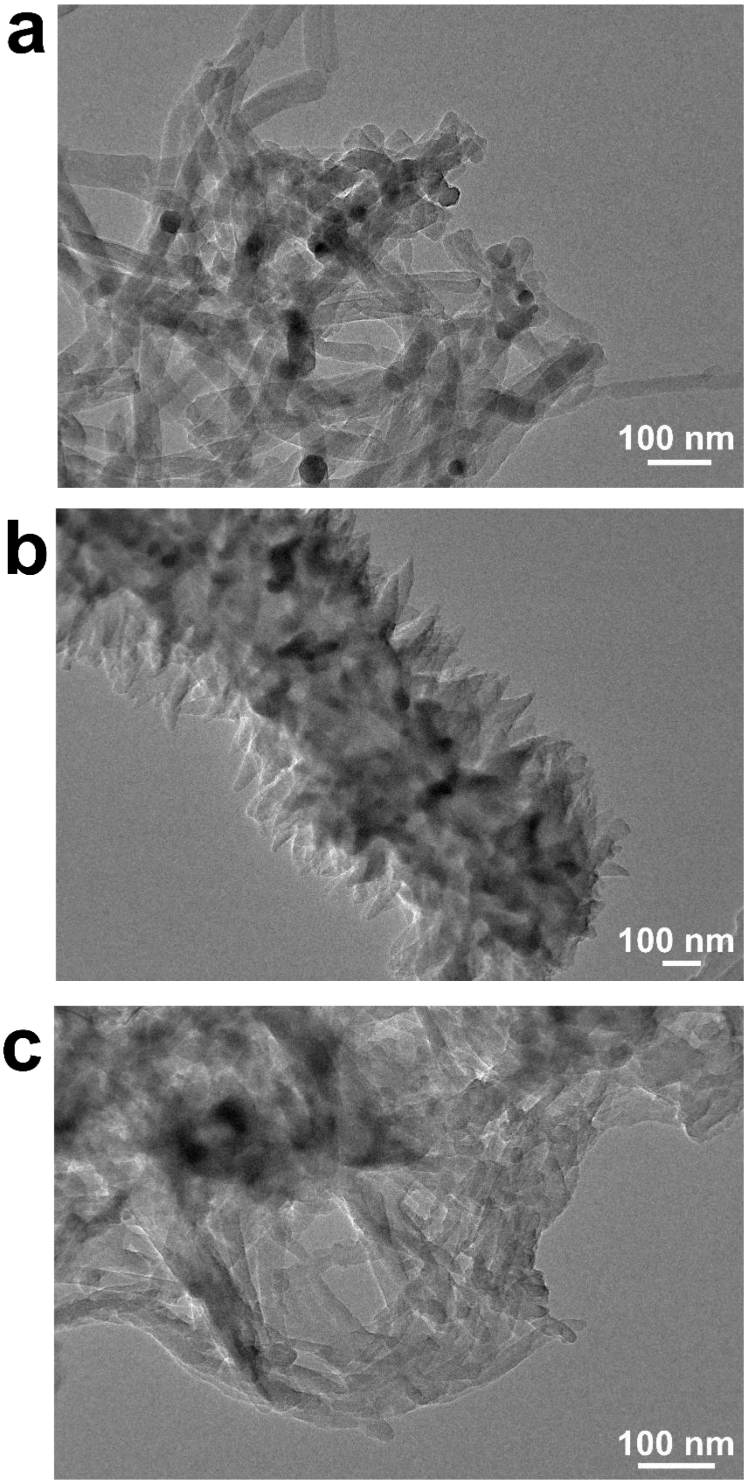

Supplementary Fig. 15 TEM images of a) MPCOF, b) OHMMCOF- DTAB, and c) OHMMCOF-OTAB.

Supplementary Section 13. EDS Mappings of MPCOF and OHMMCOF-DTAB/OHMMCOF-OTAB

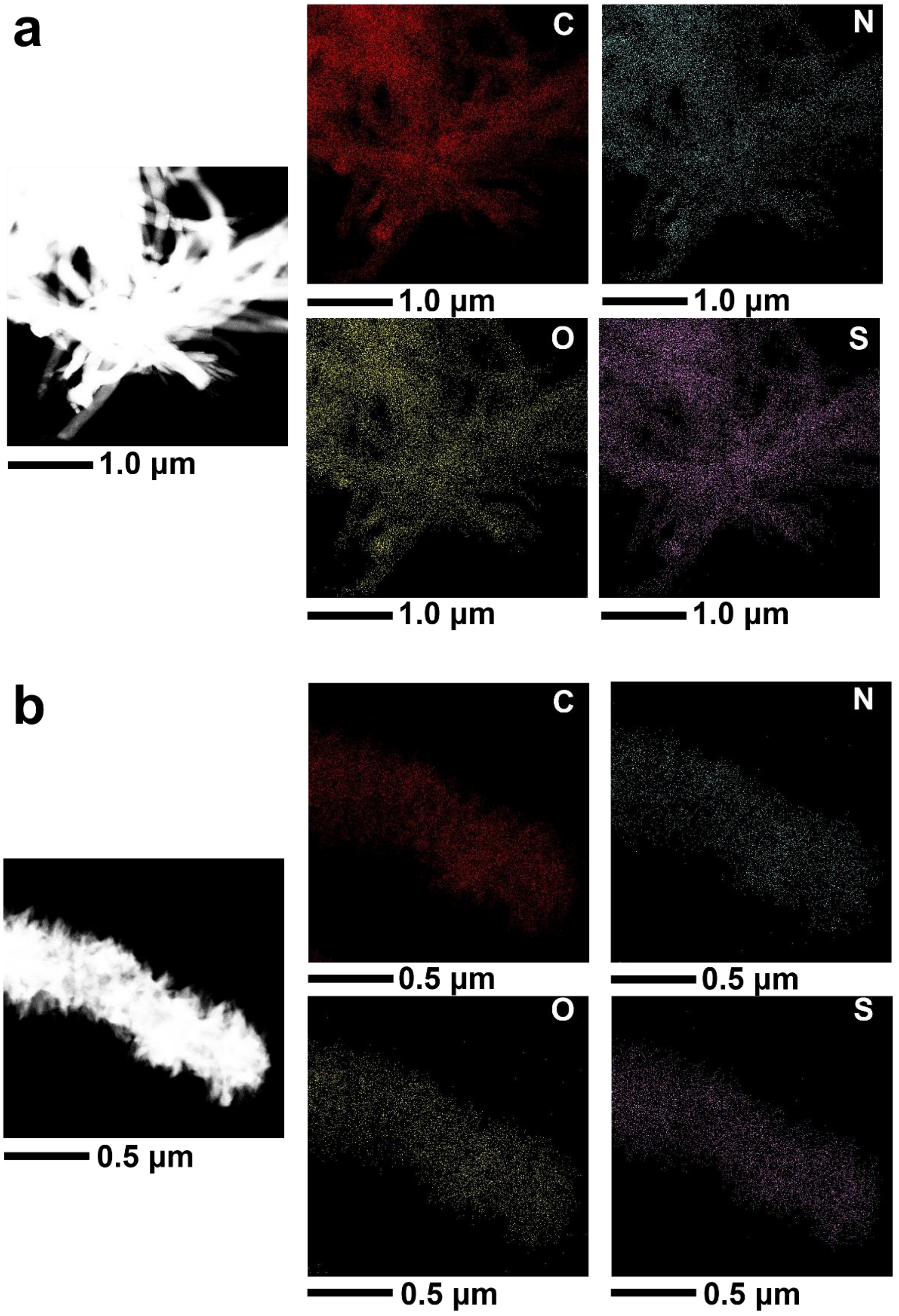

3

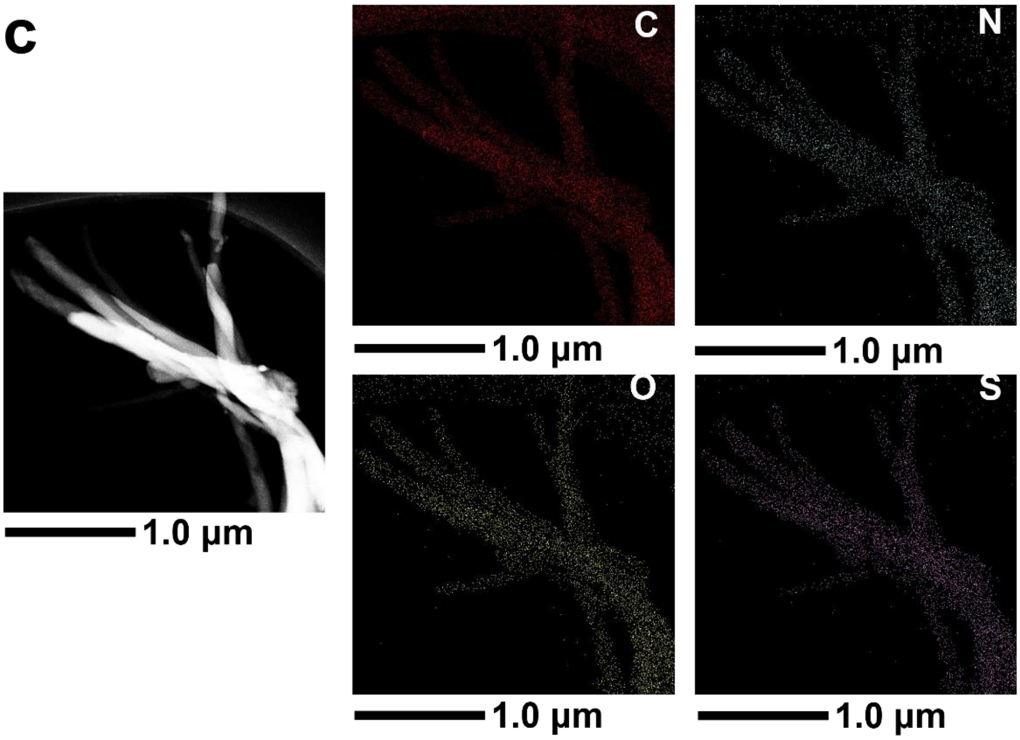

Supplementary Fig. 16 EDS mappings of a) MPCOF, b) OHMMCOF-DTAB, and c) OHMMCOF-OTAB.

Supplementary Section 14. Optimization of Experimental Conditions for Template Removal

The experiment of template removal was conducted by using the ion exchange method under certain conditions, utilizing H^+ exchange template cations. Specifically, 500 mg of activated OHMMCOF-DTAB/OHMMCOF-OTAB was put into 250 mL of H_2O/ethanol solution containing a certain concentration of hydrochloric acid. The mixture was stirred at a certain temperature for an appropriate period, and then collected by vacuum filtration, followed by H_2O and methanol washing, respectively. After vacuum drying, OHMMCOF-1/OHMMCOF-2 with ordered hierarchical micropores/mesopores was obtained. Firstly, a detailed conditional optimization experiment was carried out for the template removal experiment of OHMMCOF-DTAB (**Supplementary Fig. 17**), and then the template removal experimental parameters of OHMMCOF-OTAB was further optimized at 60 °C for 1 d (**Supplementary Fig. 18**), and PXRD and FT-IR were used to monitor the retention of crystallinity and the removal of the template, respectively.

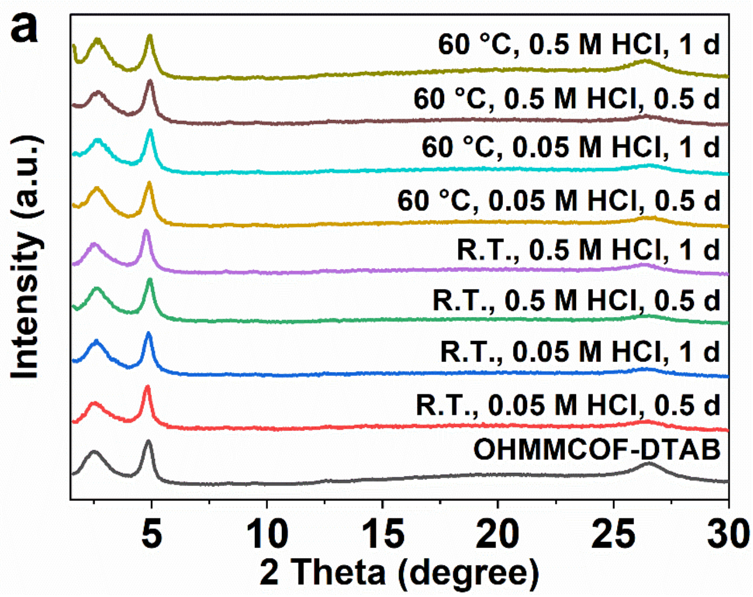

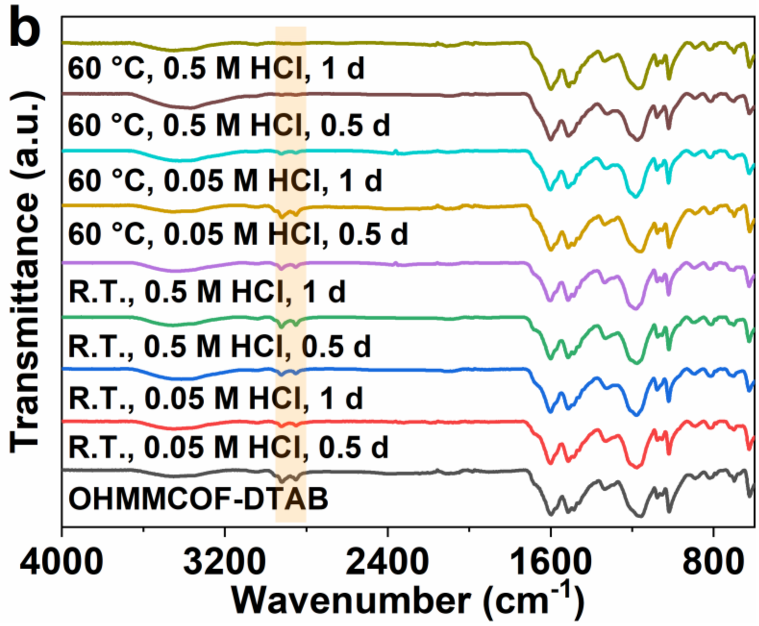

Supplementary Fig. 17 a) PXRD patterns and b) FT-IR spectra after template removal experiment on OHMMCOF-DTAB under different conditions.

The PXRD patterns of the template removal experiment of OHMMCOF-DTAB showed that the crystallinity of the material can be effectively maintained under different conditions. The FT-IR spectra indicated that as the temperature, concentration of hydrochloric acid, and duration of template removal increased, the efficiency of template removal also improved. Ultimately, DTAB was removed by stirring for 1 d in an aqueous solution of 0.5 M HCl at 60 °C.

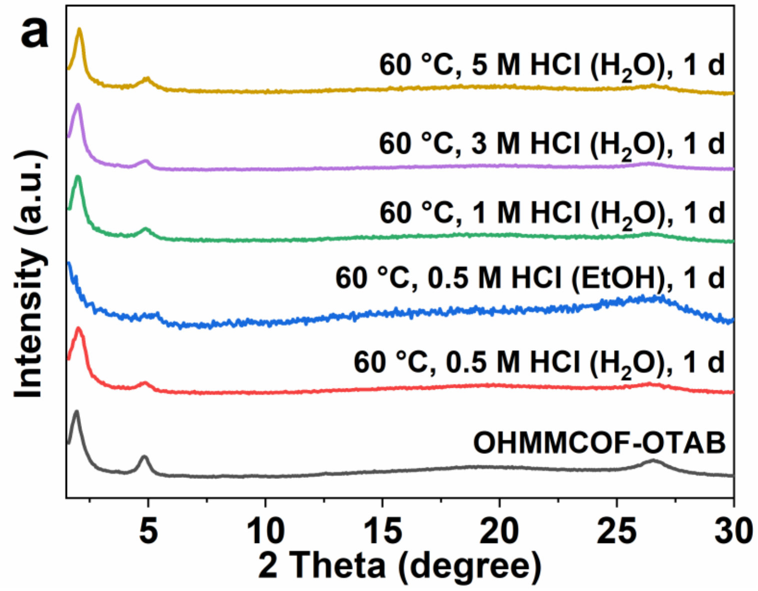

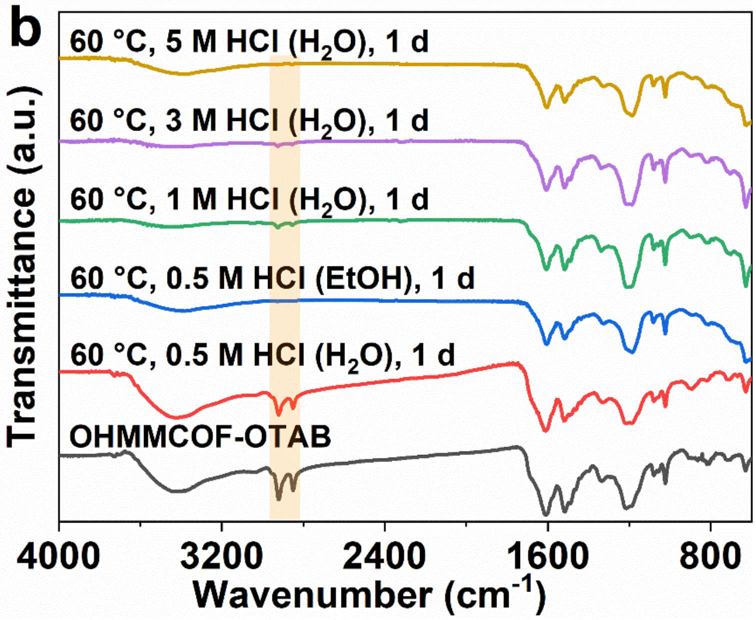

Supplementary Fig. 18 a) PXRD patterns and b) FT-IR spectra after template removal experiment on OHMMCOF-OTAB under different conditions.

The PXRD patterns and FT-IR spectra of the template removal experiment of OHMMCOF-OTAB showed that although the use of ethanol solution with a low concentration of hydrochloric acid could achieve the removal of the template, it could not maintain the crystallinity of the material. To preserve the crystallinity of OHMMCOF-OTAB, an aqueous solution of hydrochloric acid was utilized instead. The removal efficiency of the template gradually increased with the concentration of hydrochloric acid. Eventually, after stirring for 1 day in an aqueous solution of 5 M HCl at 60 °C, OTAB was completely removed from the material.

Supplementary Section 15. XPS Spectra of OHMMCOF-1/OHMMCOF-2

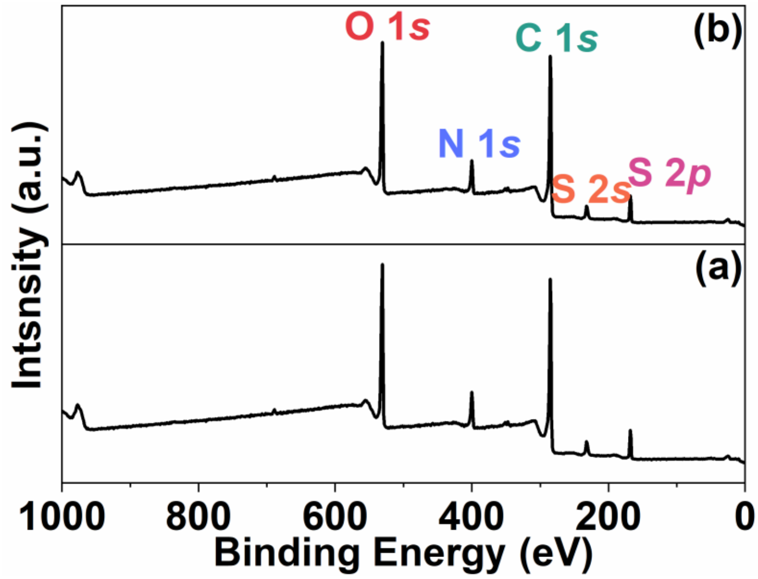

Supplementary Fig. 19 Typical XPS survey spectra of a) OHMMCOF-1, and b) OHMMCOF-2.

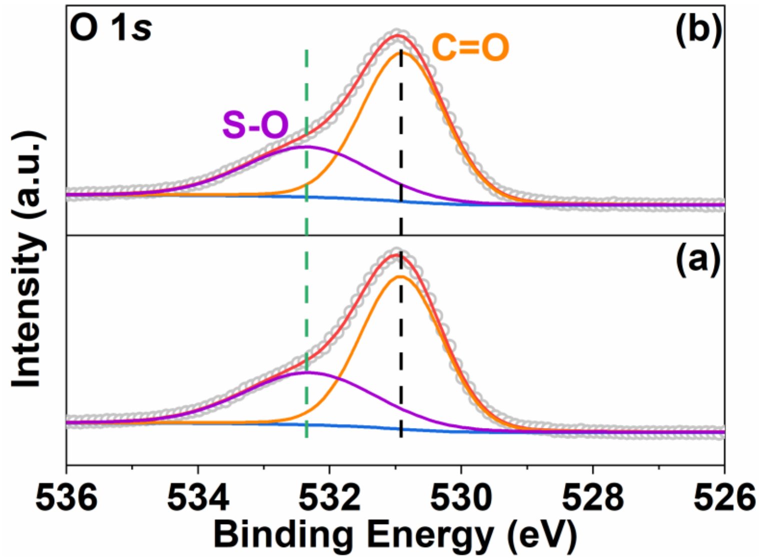

Supplementary Fig. 20 High-resolution XPS spectra of O 1*s* of a) OHMMCOF-1 and b) OHMMCOF-2.

Supplementary Section 16. BET Surface Area Plots and Pore Volume Parameters of OHMMCOF-1/OHMMCOF-2

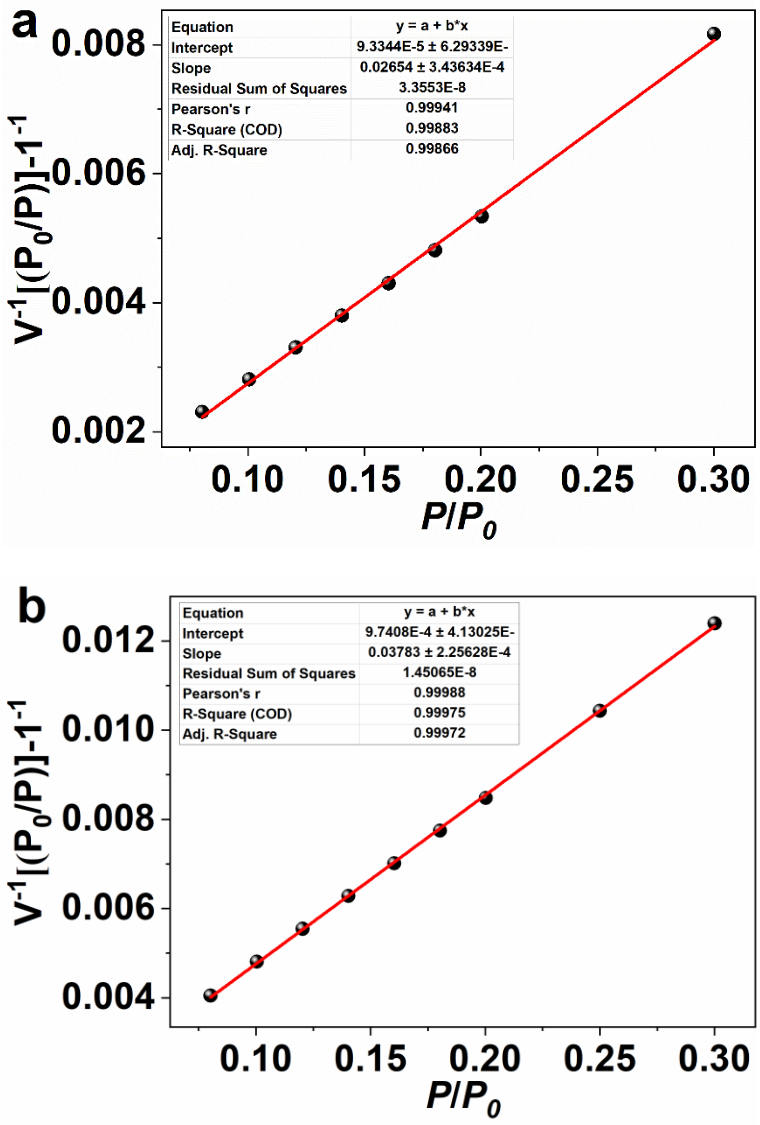

Supplementary Fig. 21 BET surface area plots of a) OHMMCOF-1 and b) OHMMCOF-2.

Supplementary Table 3. Pore volume parameters of MPCOF and OHMMCOF-DTAB/OHMMCOF-OTAB.

COF	V_{total} (cm^3/g)	$S_{BET\text{-}micro}$ (m^2/g)	$S_{BET\text{-}meso}$ (m^2/g)	$S_{BET\text{-}meso}$/ $S_{BET\text{-}micro}$
OHMMCOF-1	0.61	21.8	149.0	6.8
OHMMCOF-2	0.45	10.8	101.4	9.4

Supplementary Section 17. EA of OHMMCOF-1/OHMMCOF-2

Supplementary Table 4. EA results of OHMMCOF-1/OHMMCOF-2 (Addition of water in molecular formula may originate in adsorbed water, or in unreacted functionalities at defect sites).[5]

COF	Chemical Formula		C/ wt%	H/ wt%	N/ wt%	O/ wt%	S/ wt%
OHMMCOF-1	$C_{36}H_{30}N_6O_{11}S_3\cdot(H_2O)_7$	Theoretical values	45.76	4.69	8.89	30.48	10.18
		Experimental values	45.59	4.18	8.84	31.44	10.03
OHMMCOF-2	$C_{36}H_{30}N_6O_{11}S_3\cdot(H_2O)_7$	Theoretical values	45.76	4.69	8.89	30.48	10.18
		Experimental values	46.24	4.21	8.87	30.70	9.98

Supplementary Section 18. Water Contact Angles of OHMMCOF-1/OHMMCOF-2

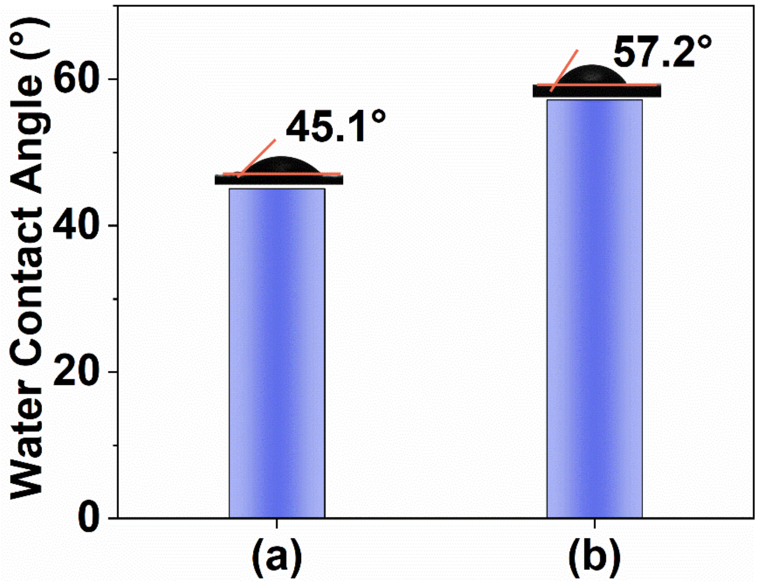

Supplementary Fig. 22 Water contact angles of a) OHMMCOF-1 and b) OHMMCOF-2.

Supplementary Section 19. TGA curves of OHMMCOF-1/OHMMCOF-2

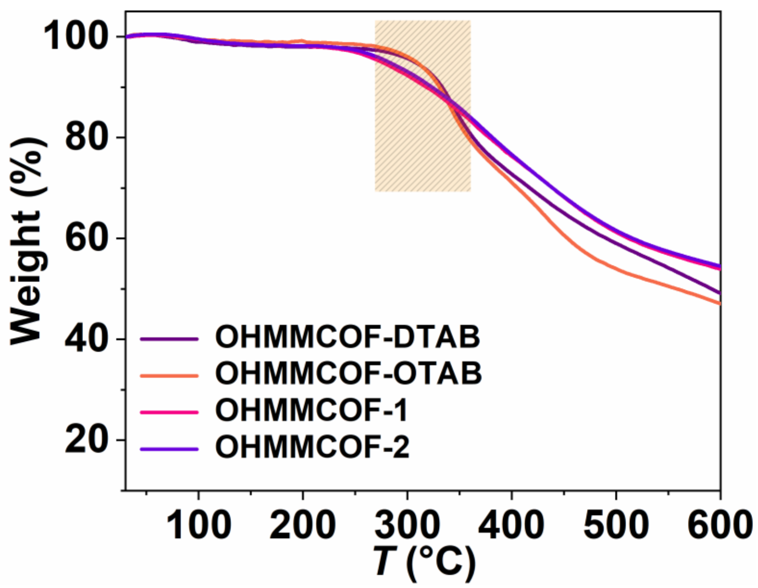

Supplementary Fig. 23 TGA curves of OHMMCOF-DTAB/OHMMCOF-OTAB and OHMMCOF-1/OHMMCOF-2.

Supplementary Section 20. SEM Images of OHMMCOF-1/OHMMCOF-2

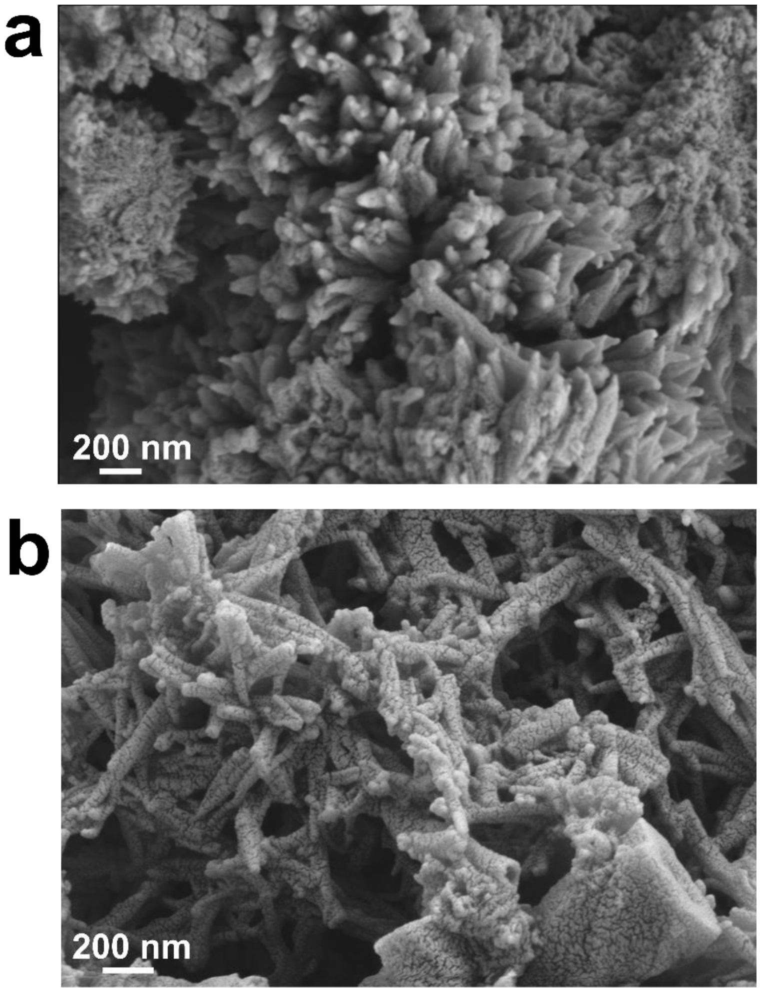

Supplementary Fig. 24 SEM images of a) OHMMCOF-1 and b) OHMMCOF-2.

Supplementary Section 21. TEM Images of OHMMCOF-1/OHMMCOF-2

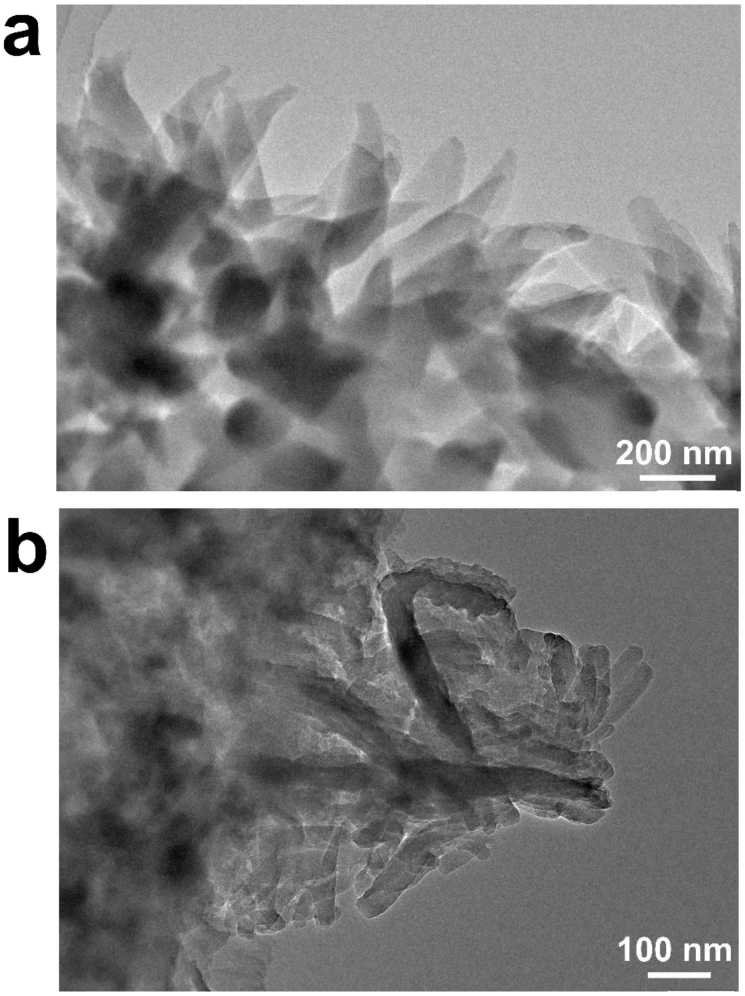

Supplementary Fig. 25 TEM images of a) OHMMCOF-1 and b) OHMMCOF-2.

Supplementary Section 22. EDS Mappings of OHMMCOF-1/OHMMCOF-2

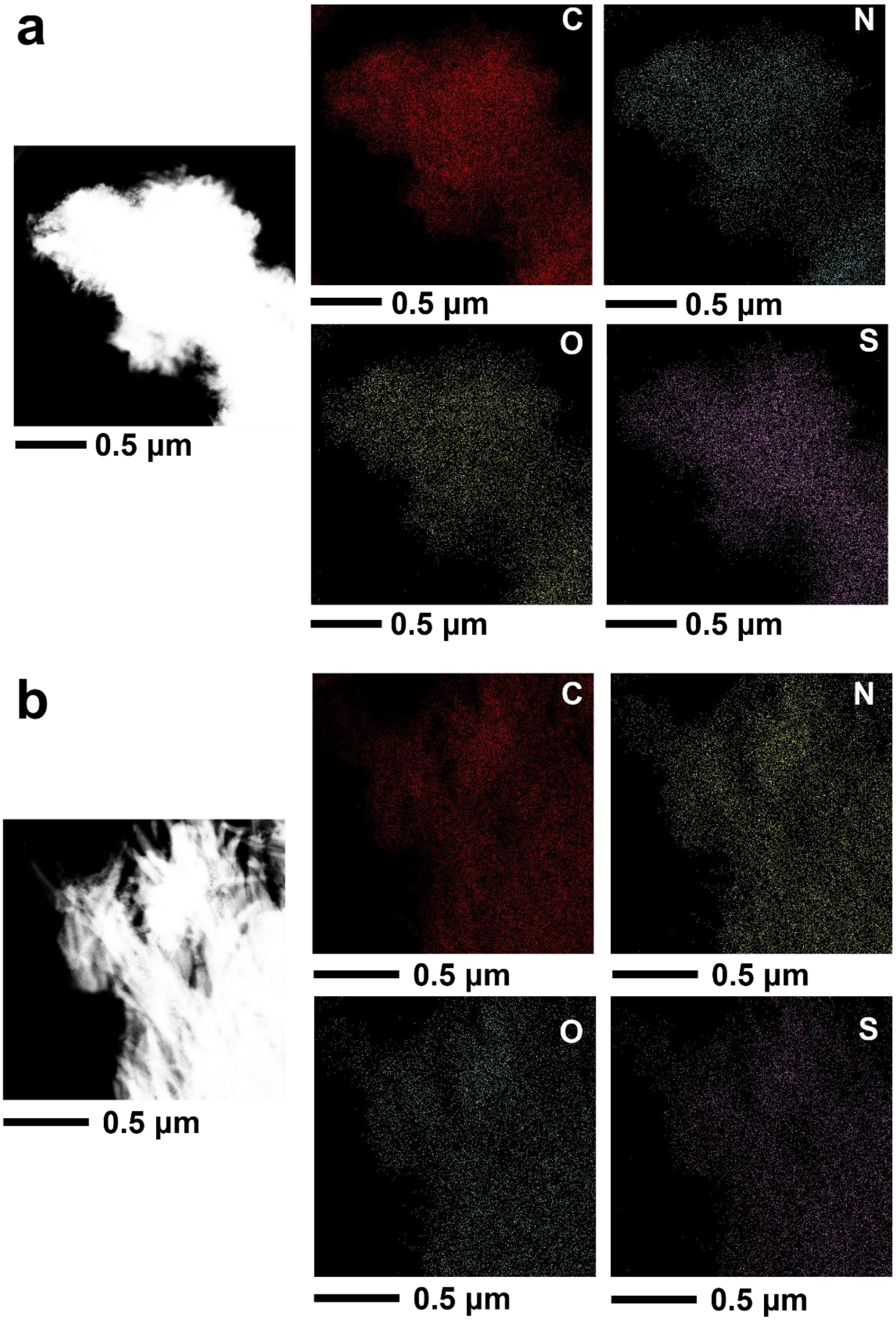

Supplementary Fig. 26 EDS mappings of a) OHMMCOF-1 and b) OHMMCOF-2.

Supplementary Section 23. HRTEM Images of OHMMCOF-1/OHMMCOF-2

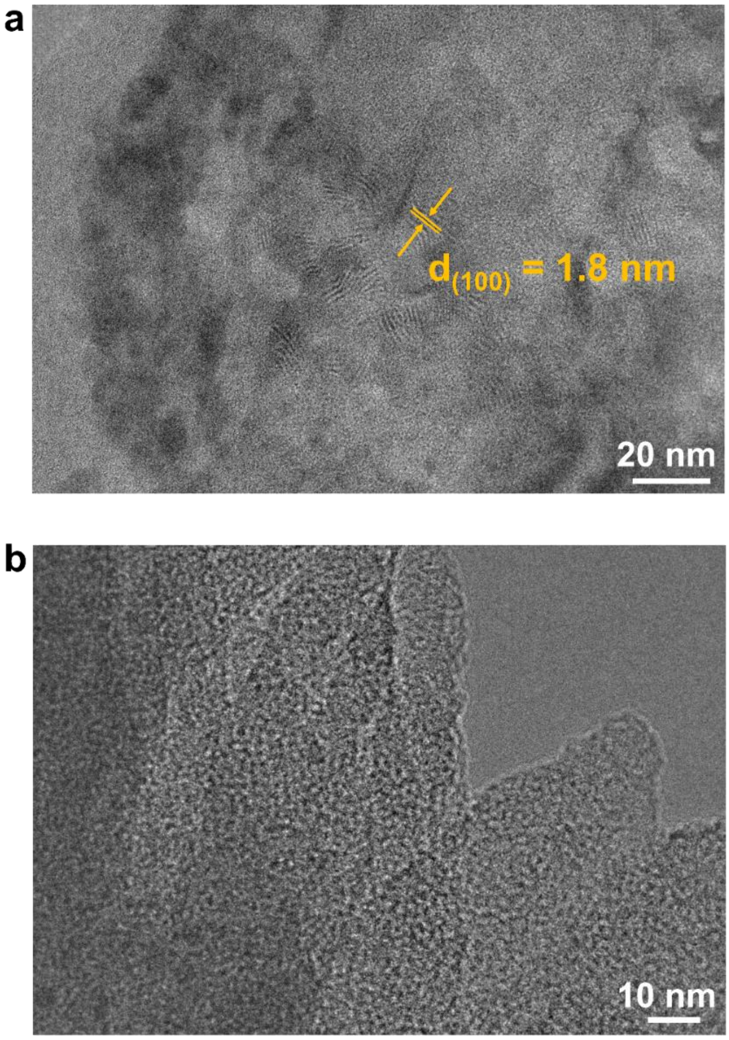

Supplementary Fig. 27 The HRTEM images for OHMMCOF-1: (a) scale bar: 20 nm (inset: (100) lattice planes), and (b) scale bar: 10 nm.

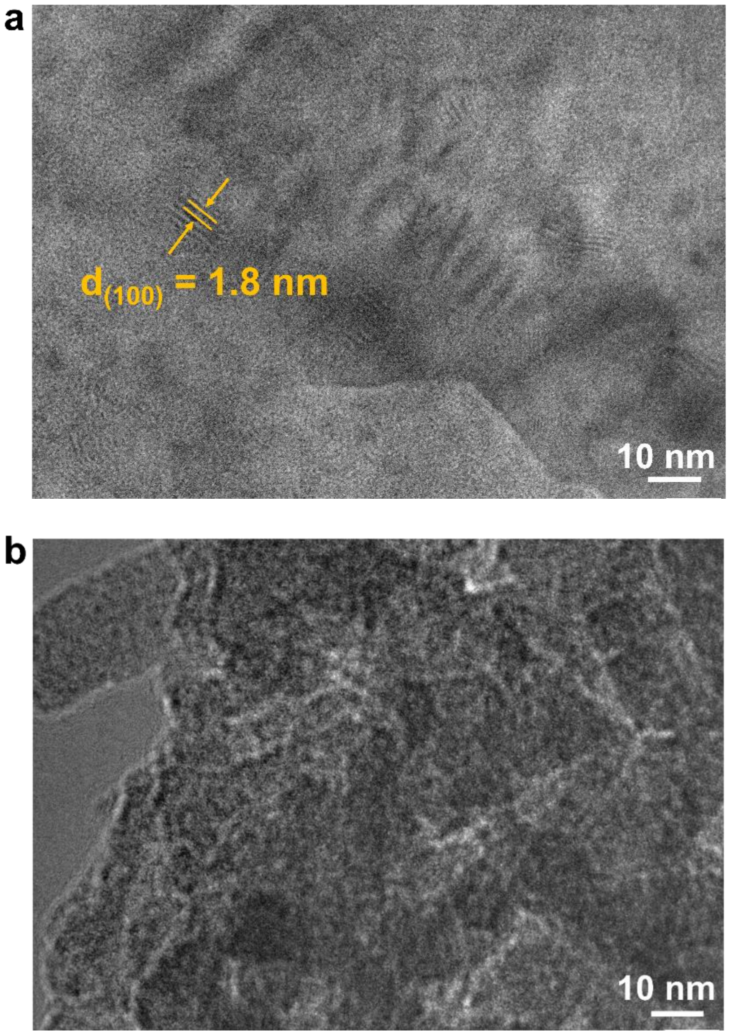

Supplementary Fig. 28 The HRTEM images for OHMMCOF-2: (a) scale bar: 10 nm (inset: (100) lattice planes), and (b) scale bar: 10 nm.

Supplementary Section 24. Influence of Template Concentration on Experimental Results

Supplementary Table 5. Input concentration and reaction concentration of different templates of OHMMCOF-DTAB/OHMMCOF-OTAB. When calculating, the monomers involved in the construction of the COF backbone in the default product are consistent with MPCOF.

OHMMCOF-DTAB		OHMMCOF-OTAB	
Input concentration (mmol)	Reaction concentration (mmol)	Input concentration (mmol)	Reaction concentration (mmol)
0.1875	0.08	0.1875	0.12
0.375	0.15	0.375	0.15
0.75	0.38	0.75	0.33
1.5	0.83	1.5	0.34
2.25	1.37	2.25	0.34

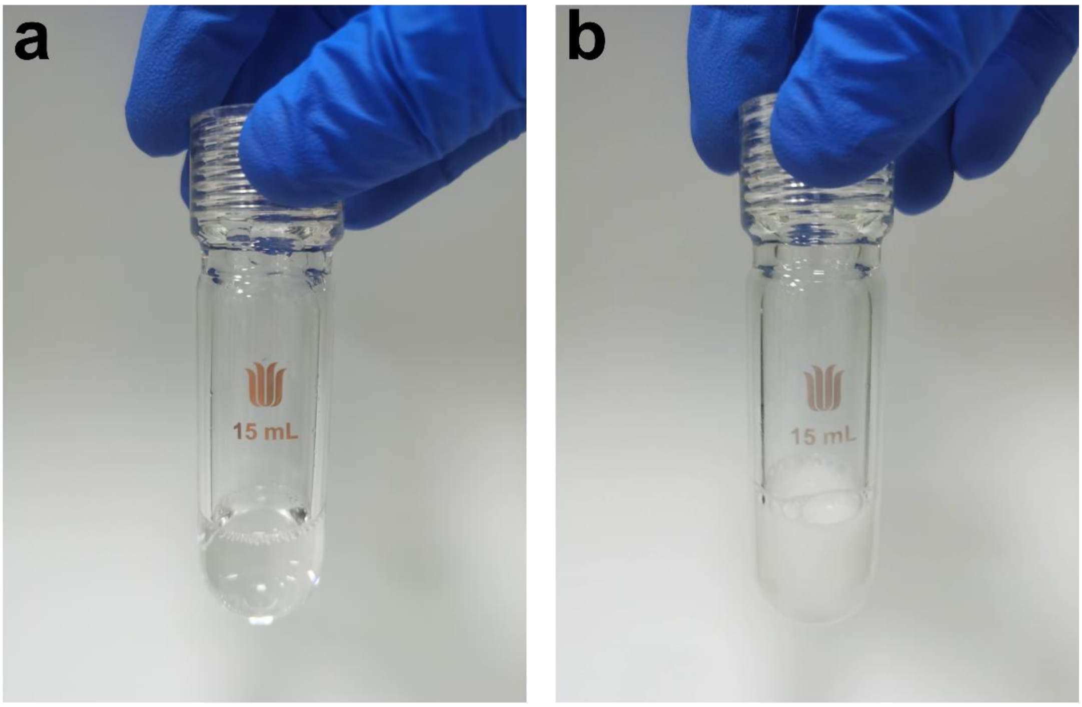

Supplementary Fig. 29 The solubility differences between a) DTAB and b) OTAB dispersed in water in the first step of the synthesis process after a 10-minute ultrasound treatment.

Supplementary Section 25. BET Surface Area Plots and Pore Volume Parameters of OHMMCOF-1-0.5/3.0 eq

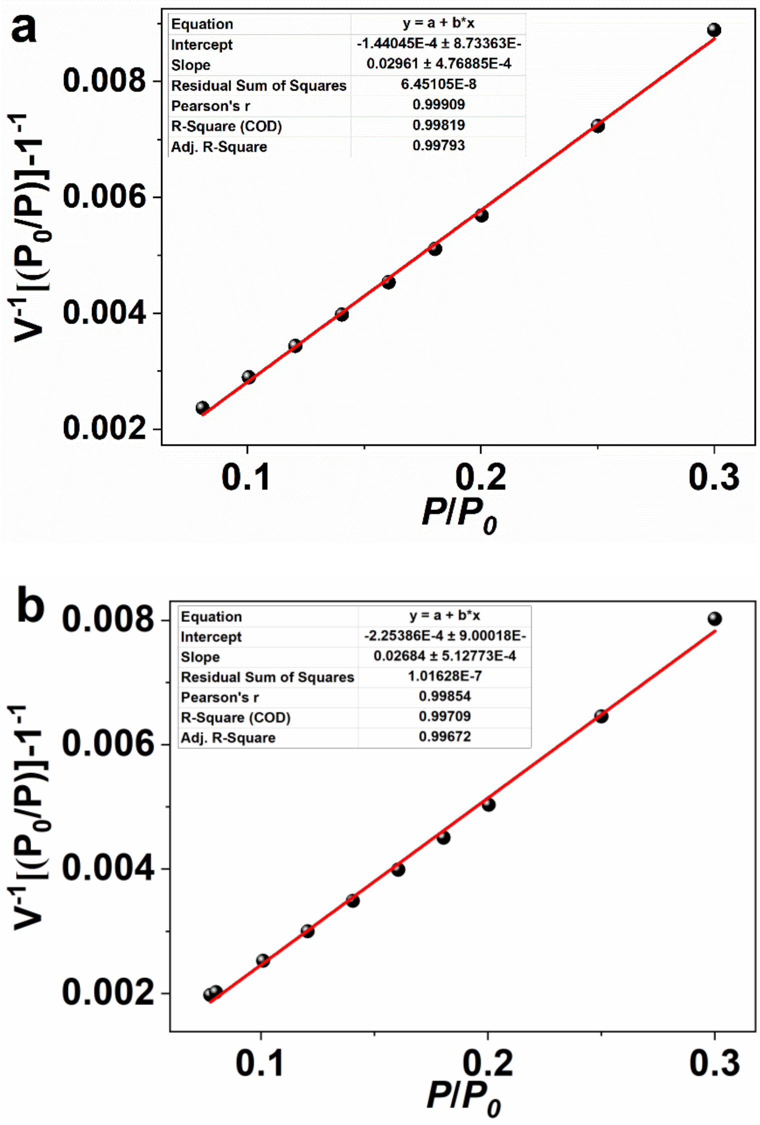

Supplementary Fig. 30 BET surface area plots of a) OHMMCOF-1-0.5 eq and b) OHMMCOF-1-3.0 eq.

Supplementary Table 6. Pore volume parameters of OHMMCOF-1-0.5/3.0 eq.

COF	V_{total} (cm^3/g)	$S_{BET\text{-}micro}$ (m^2/g)	$S_{BET\text{-}meso}$ (m^2/g)	$S_{BET\text{-}meso}$/ $S_{BET\text{-}micro}$
OHMMCOF-1-0.5 eq	0.59	24.5	134.5	5.5
OHMMCOF-1-3.0 eq	0.78	21.0	157.1	7.5

Supplementary Section 26. BET Surface Area Plots and Pore Volume Parameters of OHMMCOF-M-14:1/1:2

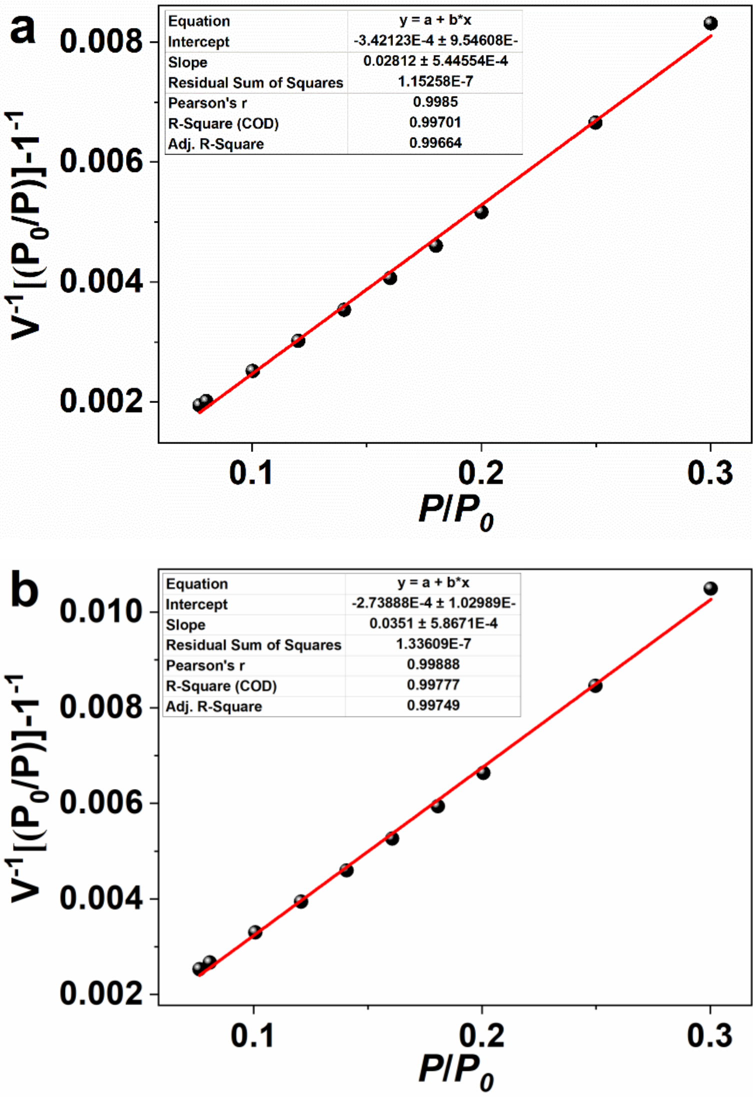

Supplementary Fig. 31 BET surface area plots of a) OHMMCOF-M-14:1 and b) OHMMCOF-M-1:2.

Supplementary Table 7. Pore volume parameters of OHMMCOF-M-14:1/1:2.

COF	V_{total} (cm^3/g)	$S_{BET\text{-}micro}$ (m^2/g)	$S_{BET\text{-}meso}$ (m^2/g)	$S_{BET\text{-}meso}$/ $S_{BET\text{-}micro}$
OHMMCOF-M-14:1	0.44	23.5	147.7	6.3
OHMMCOF-M-1:2	0.35	14.5	120.1	8.3

Supplementary Section 27. BET Surface Area Plots of OHMMCOF-3

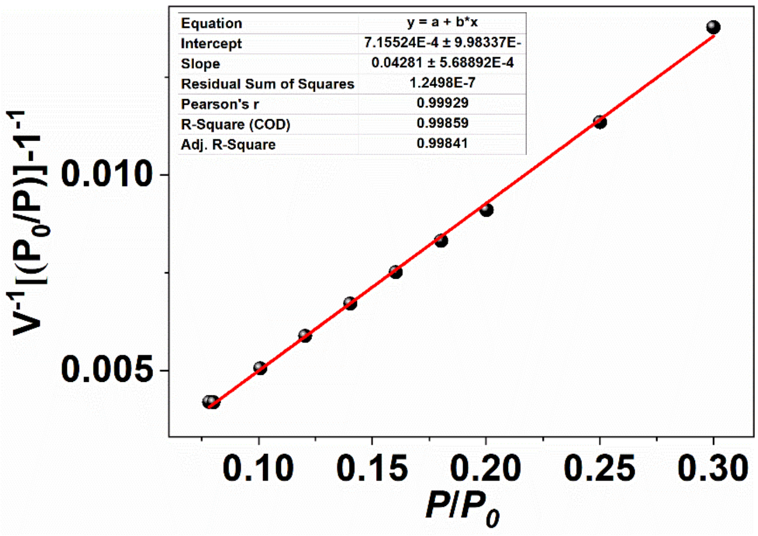

Supplementary Fig. 32 BET surface area plots of OHMMCOF-3.

Supplementary Section 28. Stability of MPCOF and OHMMCOF-1/OHMMCOF-2

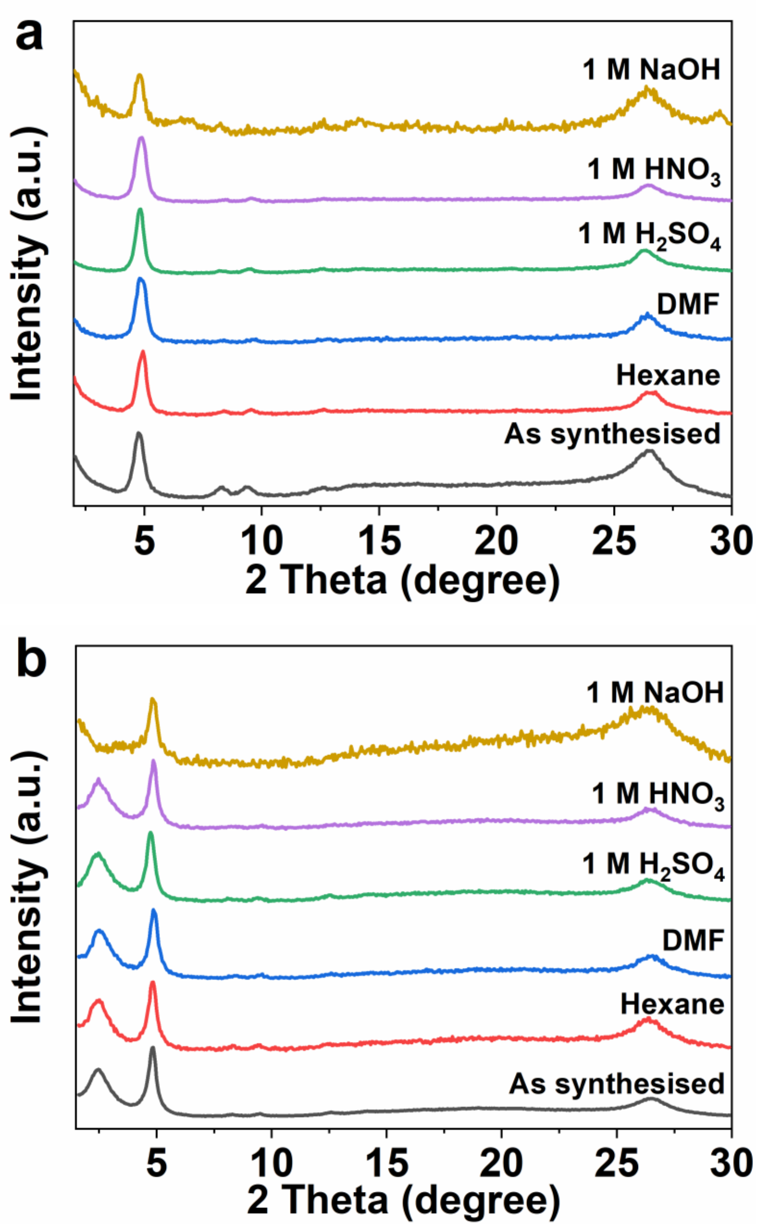

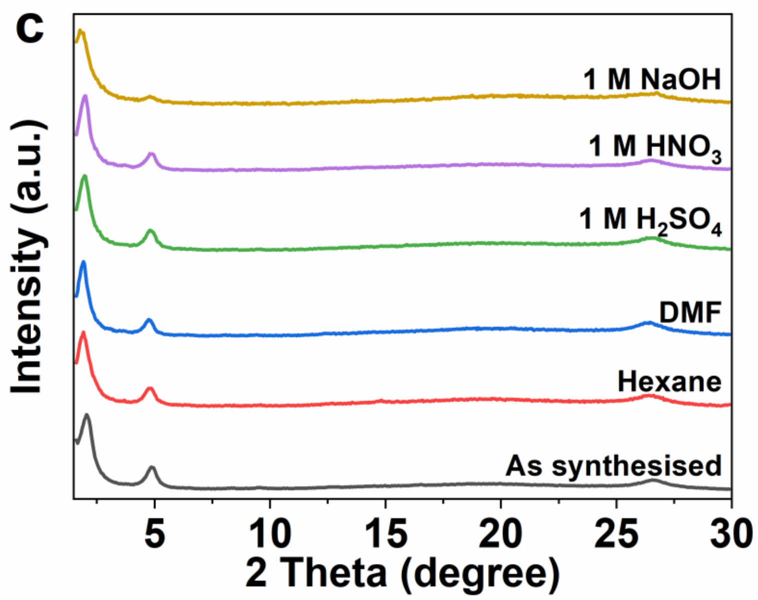

Supplementary Fig. 33 PXRD patterns of a) MPCOF, b) OHMMCOF-1, and c) OHMMCOF-2 upon 1-day treatment under different conditions.

Supplementary Section 29. Kinetic Model of MPCOF and OHMMCOF-1/OHMMCOF-2

Pseudo-first-order kinetic model:

$$\ln(q_e - q_t) = \ln q_e - k_1 t \tag{4}$$

Pseudo-second-order kinetic model:

$$\frac{t}{q_t} = \frac{1}{k_2 q_e^2} + \frac{t}{q_e} \tag{5}$$

Where q_e and q_t (mg/g) are the adsorption capacity at equilibrium and time t (h), respectively; k_1 (1/h) is the rate constant of the pseudo-first-order adsorption and k_2 (g/mg/h) is the rate constant of the pseudo-second-order adsorption. The parameters including rate constants k_1, and k_2 and correlation coefficients were calculated, and the results are given in **Supplementary Table 8-10**.

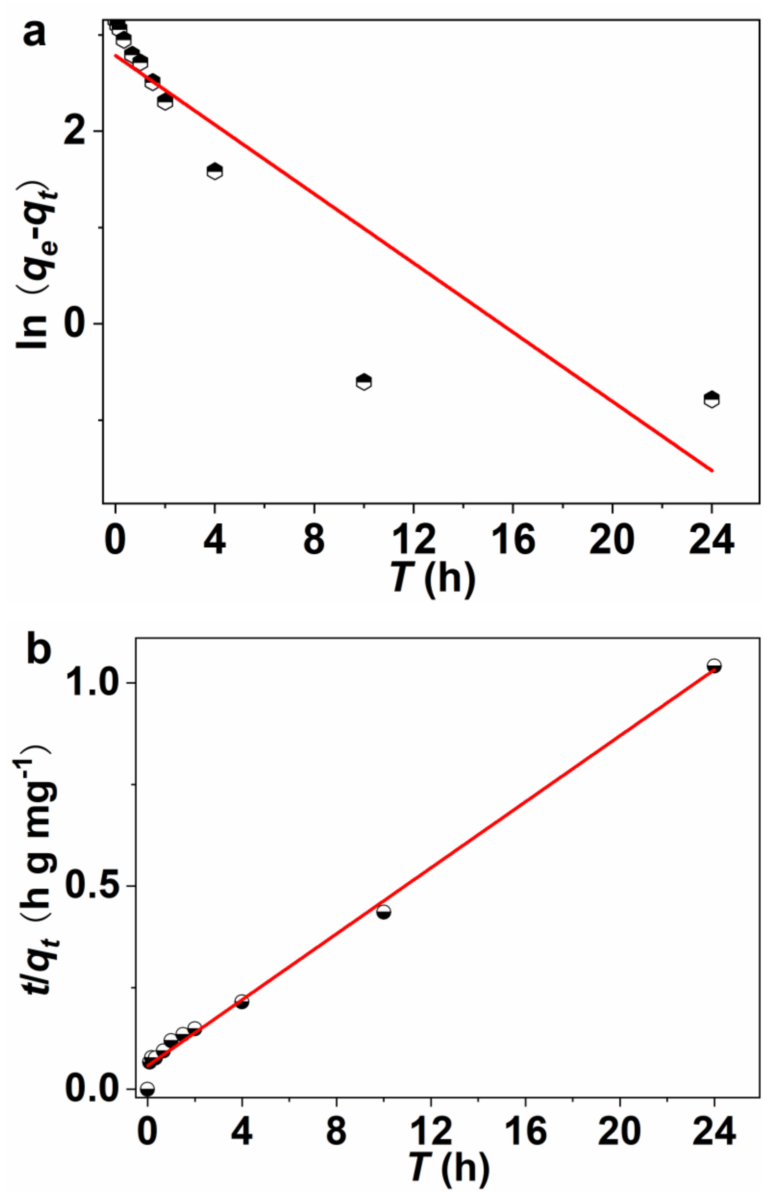

Supplementary Fig. 34 The kinetic model of MPCOF for U(VI): a) pseudo-first-order model and b) pseudo-second-order model.

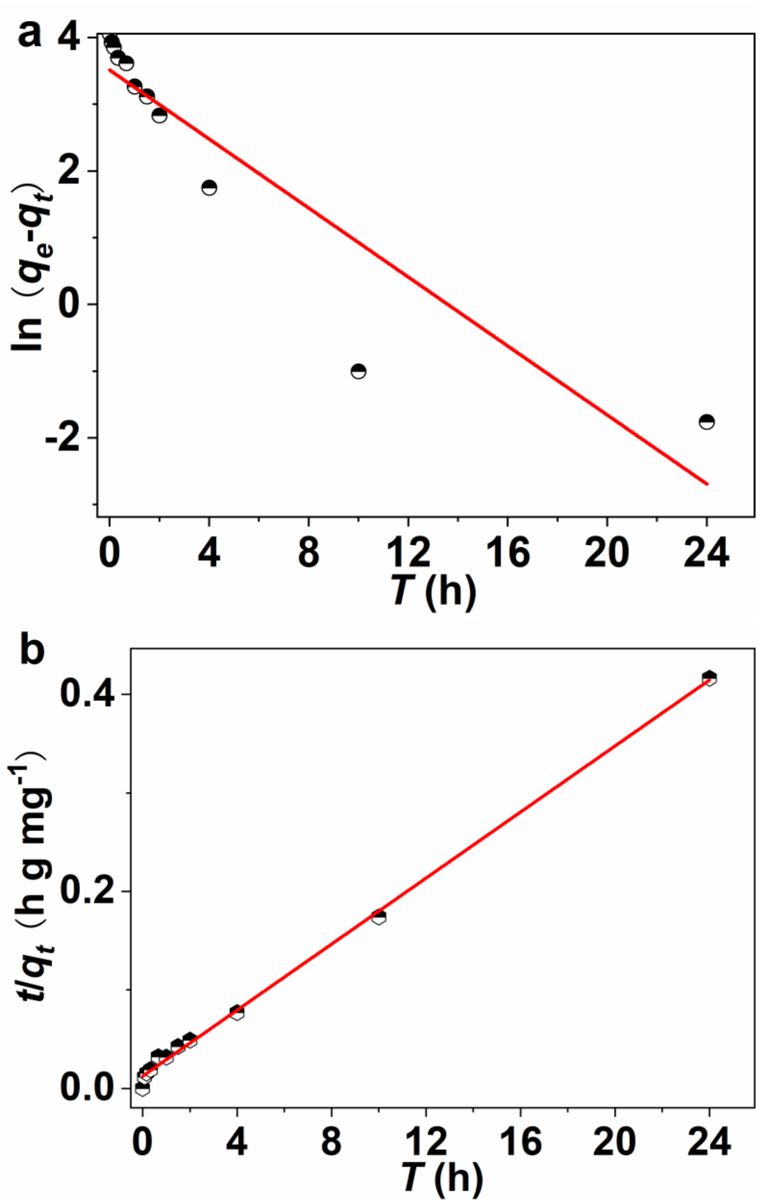

Supplementary Fig. 35 The kinetic model of MPCOF for Th(IV): a) pseudo-first-order model and b) pseudo-second-order model.

Supplementary Table 8. Parameters for kinetic models of U(VI)/Th(IV) adsorption by MPCOF.

Nuclide	$Q_e^{(exp)}$ (mg/g)	Pseudo-first-order			Pseudo-second-order		
		k_1 (1/h)	$Q_e^{(cal)}$ (mg/g)	R_1^2	k_2 (g/mg/h)	$Q_e^{(cal)}$ (mg/g)	R_2^2
U(VI)	23.0	0.17958	16.3	0.79875	0.02864	24.6	0.99319
Th(IV)	57.6	0.25841	33.6	0.8394	0.02272	59.7	0.99761

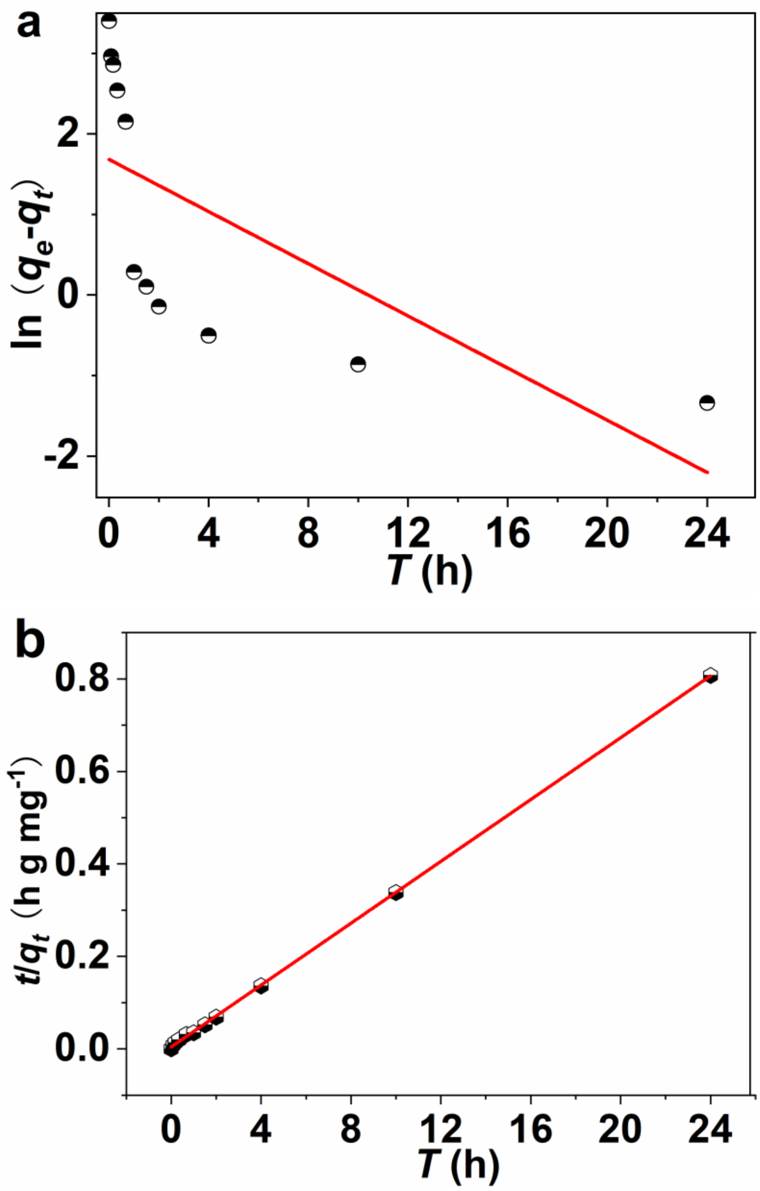

Supplementary Fig. 36 The kinetic model of OHMMCOF-1 for U(VI): a) pseudo-first-order model and b) pseudo-second-order model.

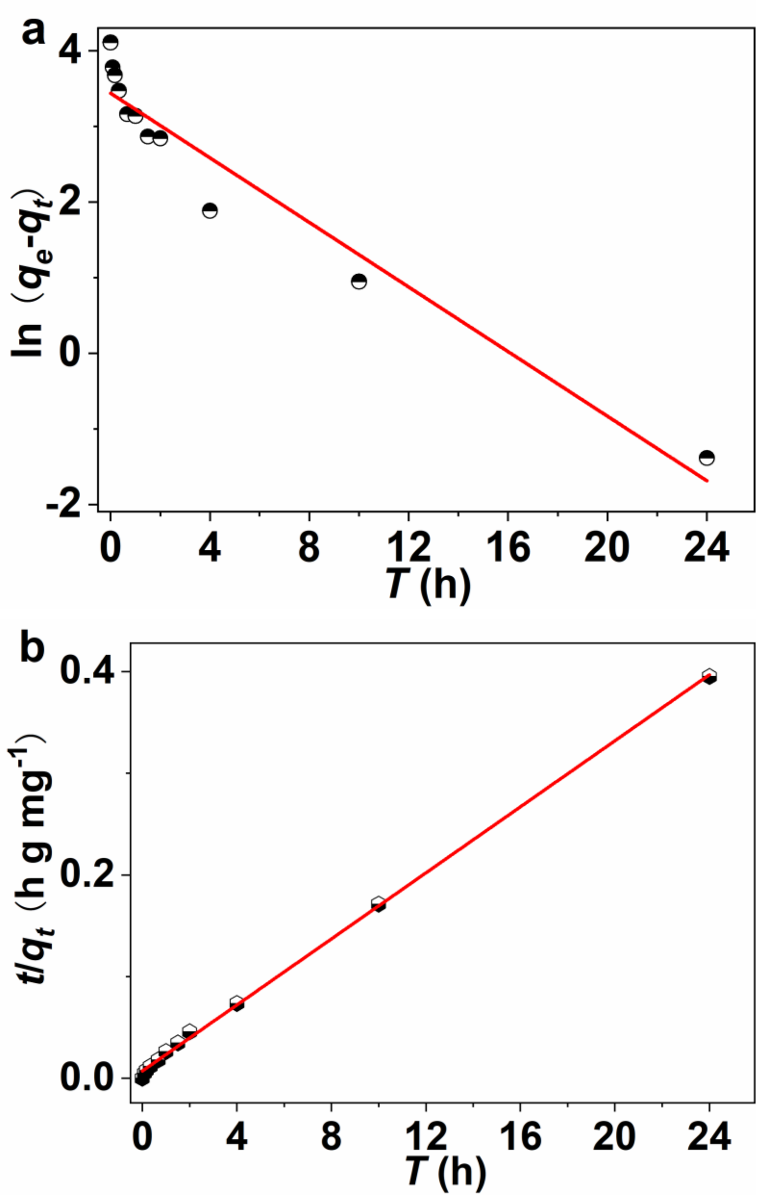

Supplementary Fig. 37 The kinetic model of OHMMCOF-1 for Th(IV): a) pseudo-first-order model and b) pseudo-second-order model.

Supplementary Table 9. Parameters for kinetic models of U(VI)/Th(IV) adsorption by OHMMCOF-1.

Nuclide	$Q_e^{(exp)}$ (mg/g)	Pseudo-first-order			Pseudo-second-order		
		k_1 (1/h)	$Q_e^{(cal)}$ (mg/g)	R_1^2	k_2 (g/mg/h)	$Q_e^{(cal)}$ (mg/g)	R_2^2
U(VI)	29.7	0.16196	5.4	0.39034	0.26806	29.9	0.99983
Th(IV)	60.8	0.21354	31.2	0.93486	0.03726	61.6	0.99895

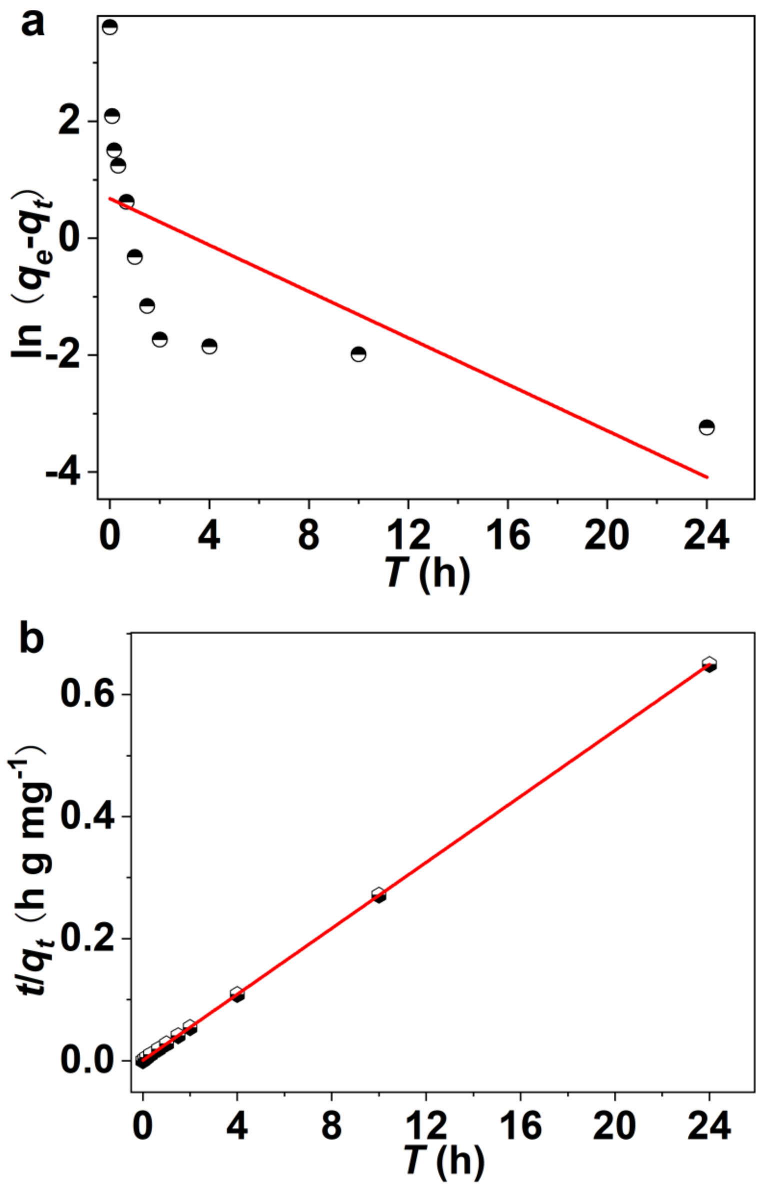

Supplementary Fig. 38 The kinetic model of OHMMCOF-2 for U(VI): a) pseudo-first-order model and b) pseudo-second-order model.

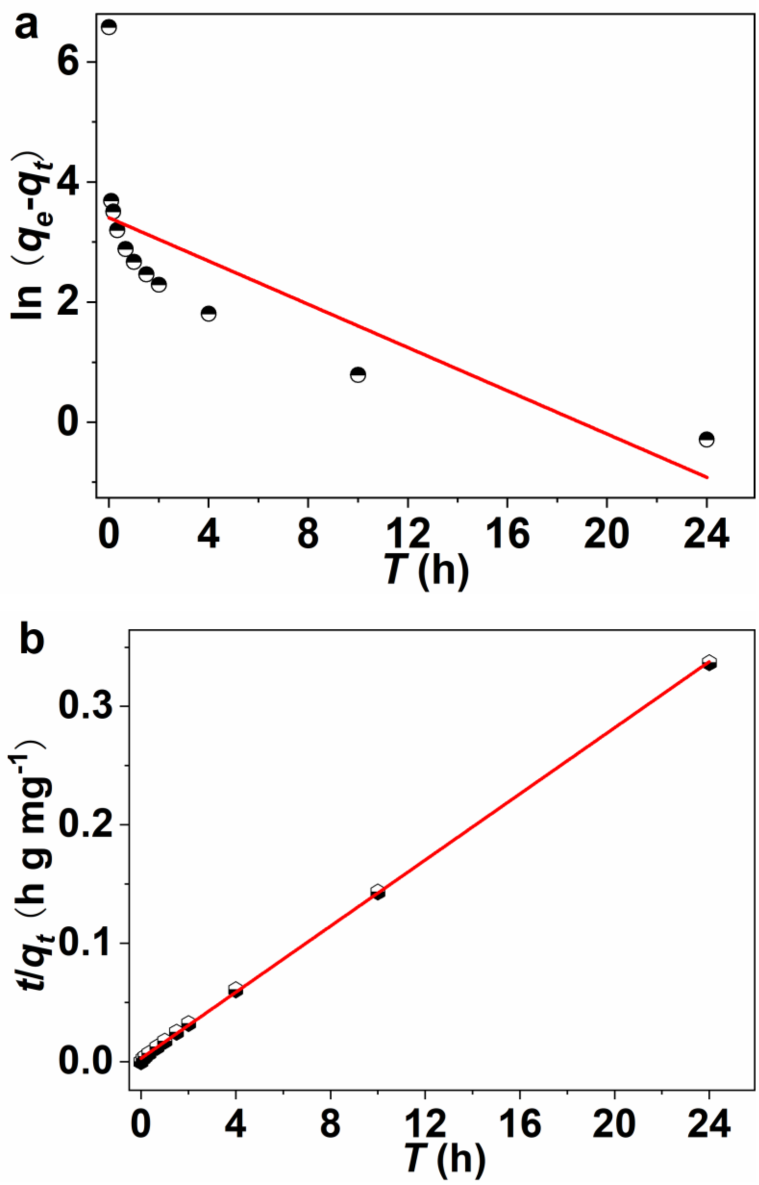

Supplementary Fig. 39 The kinetic model of OHMMCOF-2 for Th(IV): a) pseudo-first-order model and b) pseudo-second-order model.

Supplementary Table 10. Parameters for kinetic models of U(VI)/Th(IV) adsorption by OHMMCOF-2.

Nuclide	$Q_e^{(exp)}$ (mg/g)	Pseudo-first-order			Pseudo-second-order		
		k_1 (1/h)	$Q_e^{(cal)}$ (mg/g)	R_1^2	k_2 (g/mg/h)	$Q_e^{(cal)}$ (mg/g)	R_2^2
U(VI)	37.0	0.19851	2.0	0.41501	1.34610	37.0	1
Th(IV)	71.3	0.18003	32.0	0.50859	0.06942	71.6	0.99978

Supplementary Section 30. The Species Distributions of U(VI) and Th(IV) under Various Acidic Conditions

The species of U(VI)/Th(IV) under various acidic conditions were calculated by using Visual MINTEQ software (version 3.1). The variables for entering U(VI)/Th(IV) included a concentration of 50 ppm, a temperature of 25 °C, a pressure of 1 atm, and a pH in the range of 0 - 14. The results of this calculation can be found in **Supplementary Fig. 40**.

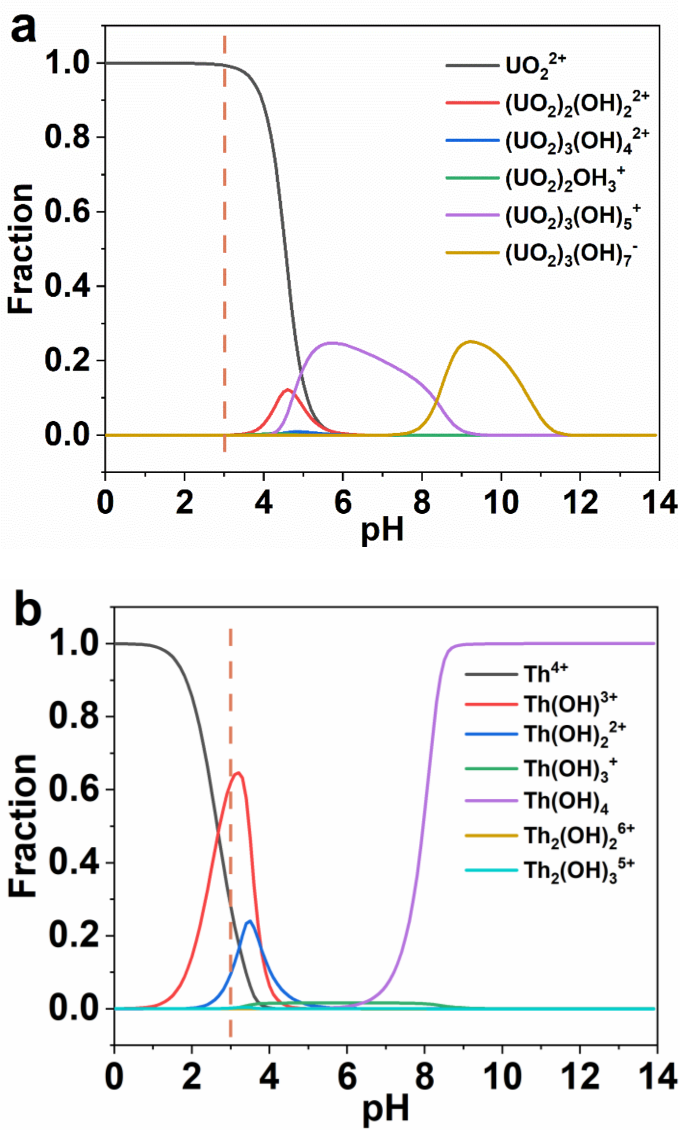

Supplementary Fig. 40 The species distributions of a) U(VI) and b) Th(IV) under various acidic conditions.

Supplementary Section 31. Experimental Results of MPCOF-Organic Synthesized by Organic Solvothermal Method

The solvothermal method was used to select trimethylbenzene and dioxane with a volume ratio of 1:1 as the synthesis solvents, and the parent COF was synthesized and named MPCOF-Organic. Subsequently, PXRD and N_2 adsorption-desorption were used to characterize the crystallinity and porosity of the material, respectively. Finally, the U(VI)/Th(IV) kinetic adsorption properties of the material were evaluated (**Supplementary Fig. 41**). MPCOF-Organic exhibited good crystallinity and higher BET and pore volume than aqueous MPCOF, which may be because water as a solvent reduces the partial reversibility of the synthesis process. The results of U(VI)/Th(IV) kinetic adsorption experiments showed that MPCOF-Organic (23.2 mg/g-U(VI) and 58.3 mg/g-Th(IV)) and MPCOF exhibited almost the same U(VI)/Th(IV) adsorption capacity, and the U(VI)/Th(IV) kinetics are relatively weakly increased (0.7 mg/(g·min)-U(VI) and 1.7 mg/(g·min)-Th(IV) in the first 5 minutes).

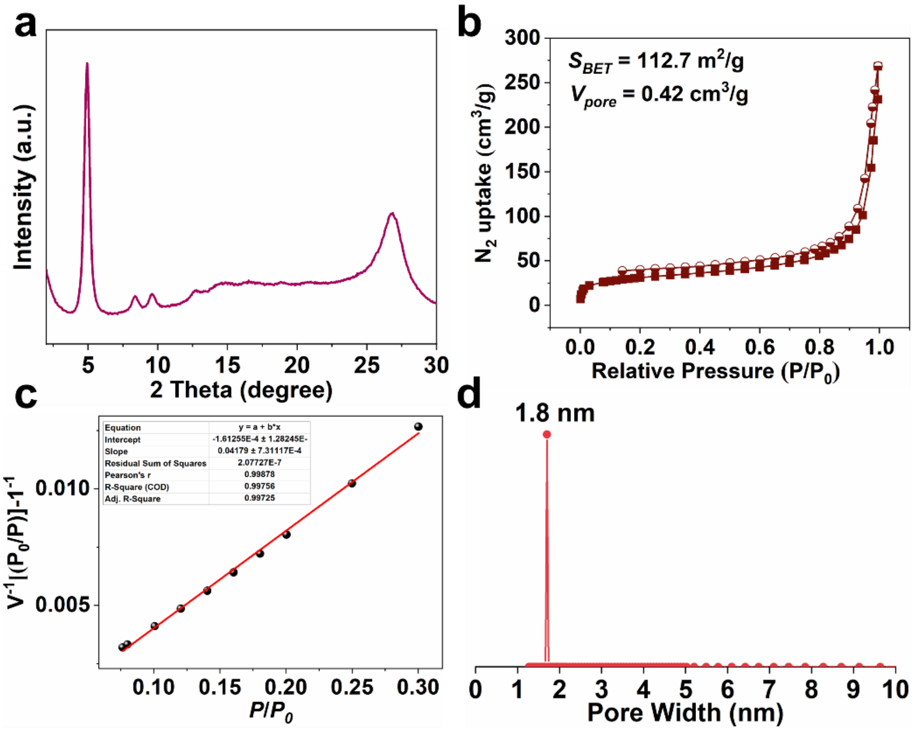

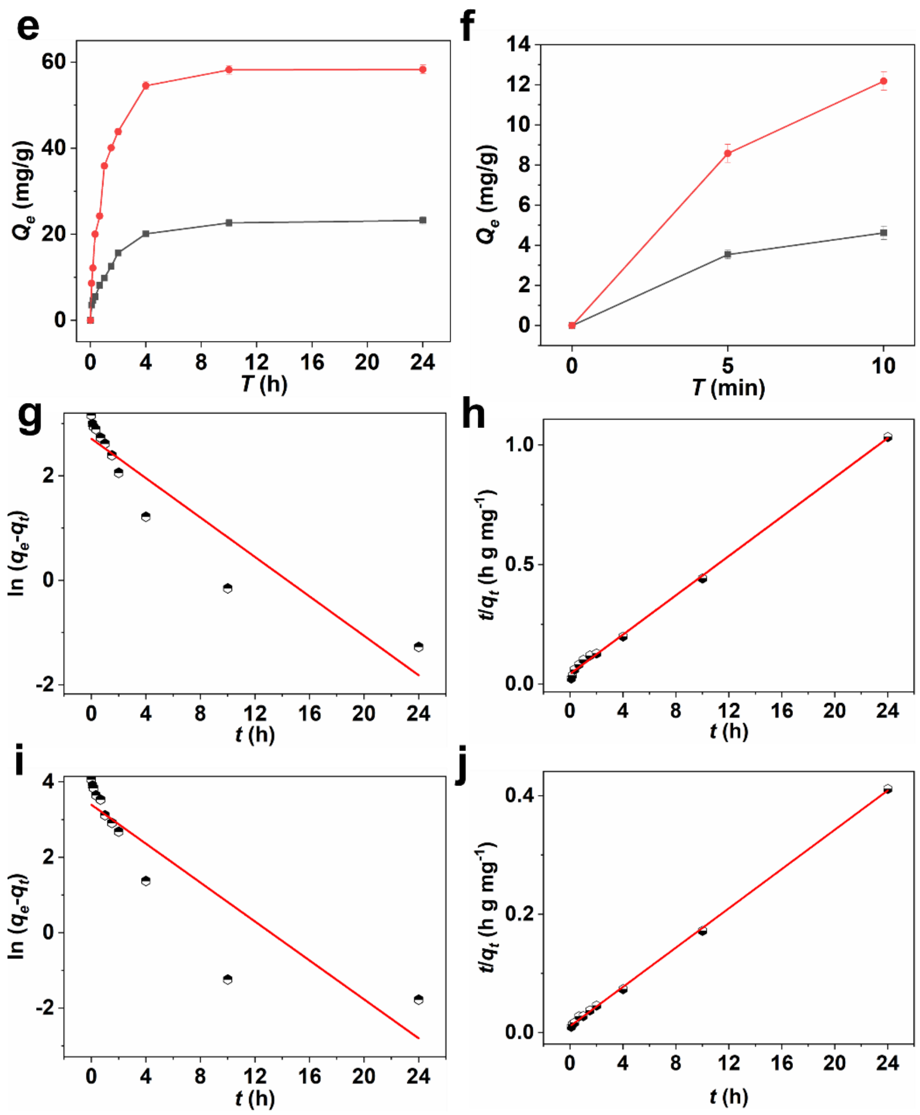

Supplementary Fig. 41. MPCOF-Organic: a) PXRD patterns, b) nitrogen adsorption-desorption isotherms at 77 K, c) BET surface area plots, d) the corresponding pore size distributions, e) adsorption kinetics at 24 h and f) 10 min, the kinetic model for U(VI): g) pseudo-first-order model and h) pseudo-second-order model, and kinetic model for Th(IV): i) pseudo-first-order model and j) pseudo-second-order model. All the error bars represent the standard deviation of the experiments.

Supplementary Table 11. Parameters for kinetic models of U(VI)/Th(IV) adsorption by MPCOF-Organic.

Nuclide	$Q_e^{(exp)}$ (mg/g)	Pseudo-first-order			Pseudo-second-order		
		k_1 (1/h)	$Q_e^{(cal)}$ (mg/g)	R_1^2	k_2 (g/mg/h)	$Q_e^{(cal)}$ (mg/g)	R_2^2
U(VI)	23.2	0.18854	15.1	0.87769	0.03971	24.3	0.99786
Th(IV)	58.3	0.25814	29.9	0.80452	0.02519	60.3	0.99929

Supplementary Section 32. Time-Dependent Kinetic Adsorption Experiments and Kinetic Model of OHMMCOF-1-U(VI)/Th(IV) with Different Template Concentrations

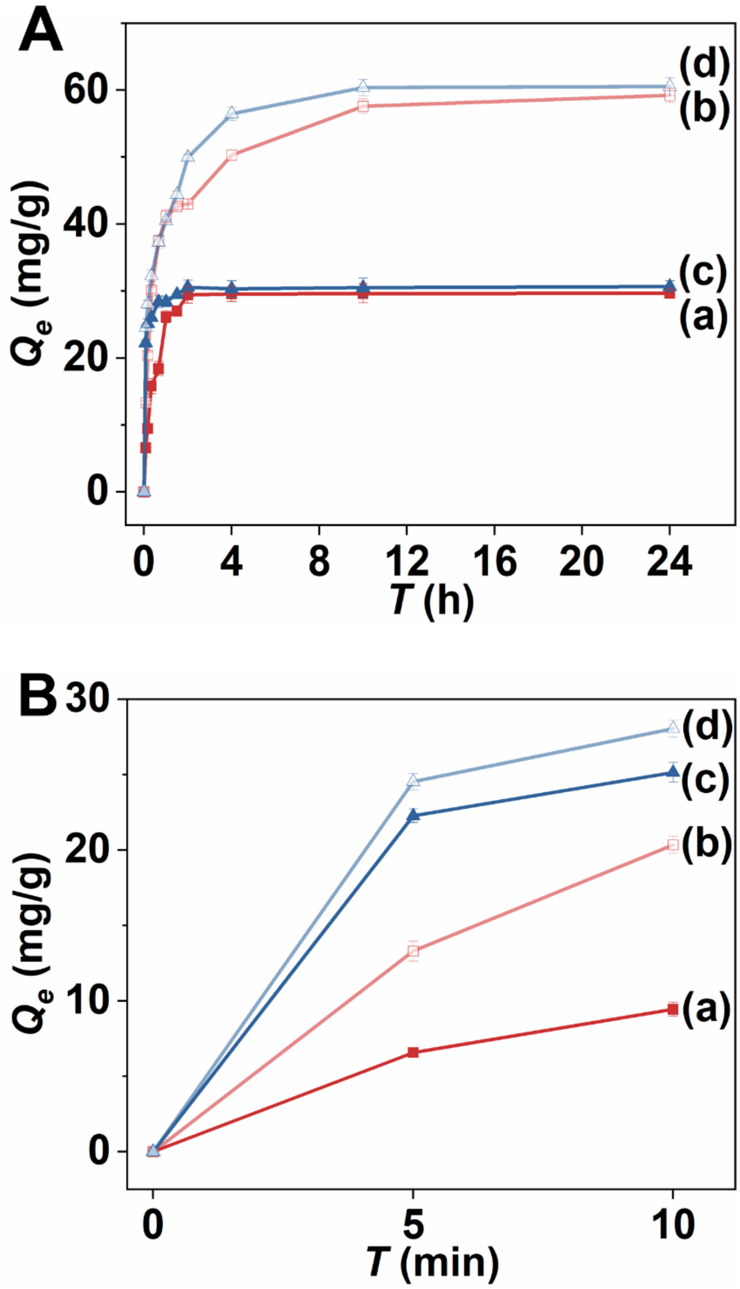

Supplementary Fig. 42 Adsorption kinetics at A) 24 h and B) 10 min ((a) OHMMCOF-1-0.5 eq-U, (b) OHMMCOF-1-0.5 eq-Th, (c) OHMMCOF-1-3.0 eq-U, and (d) OHMMCOF-1-3.0 eq-Th). All the error bars represent the standard deviation of the experiments.

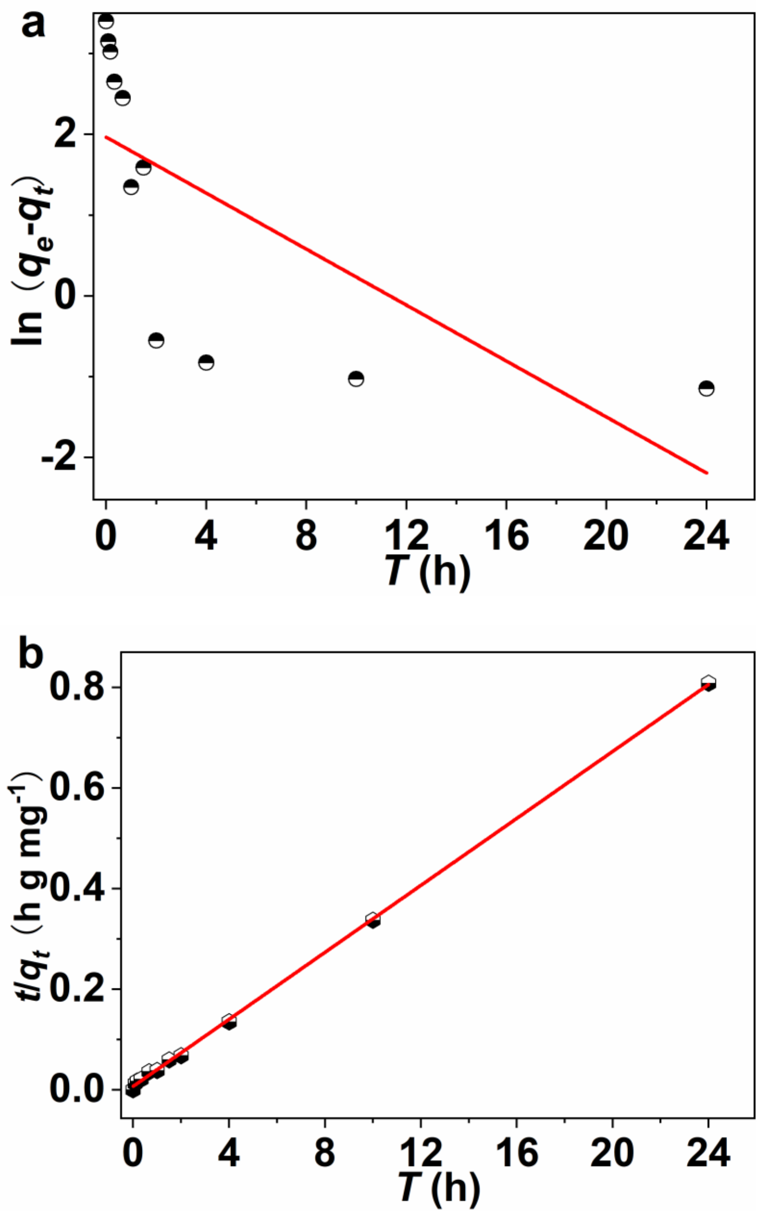

Supplementary Fig. 43 The kinetic model of OHMMCOF-1-0.5 eq as the template for U(VI): a) pseudo-first-order model and b) pseudo-second-order model.

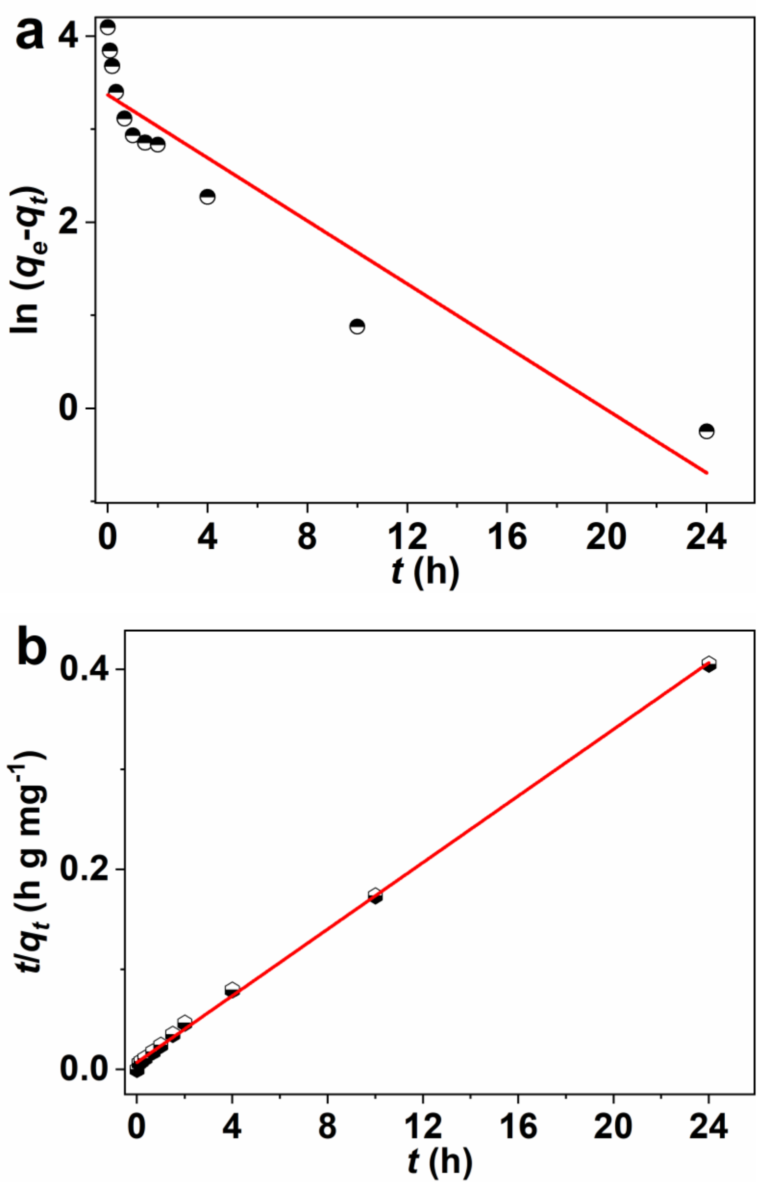

Supplementary Fig. 44 The kinetic model of OHMMCOF-1-0.5 eq as the template for Th(IV): a) pseudo-first-order model and b) pseudo-second-order model.

Supplementary Table 12. Parameters for kinetic models of U(VI)/Th(IV) adsorption by OHMMCOF-1-0.5 eq.

Nuclide	$Q_e^{(exp)}$ (mg/g)	Pseudo-first-order			Pseudo-second-order		
		k_1 (1/h)	$Q_e^{(cal)}$ (mg/g)	R_1^2	k_2 (g/mg/h)	$Q_e^{(cal)}$ (mg/g)	R_2^2
U(VI)	29.4	0.17336	7.2	0.41404	0.15739	30.0	0.99958
Th(IV)	59.2	0.16786	29.1	0.86419	0.03850	60.1	0.99890

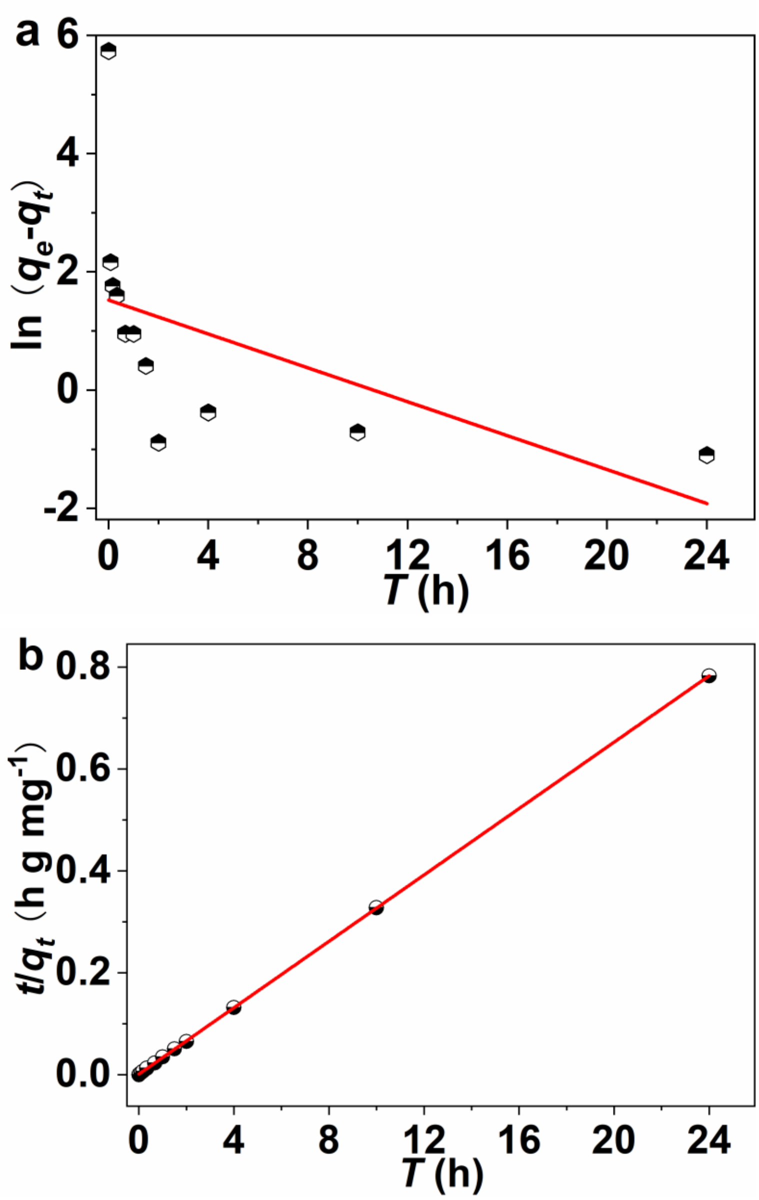

Supplementary Fig. 45 The kinetic model of OHMMCOF-1-3.0 eq as the template for U(VI): a) pseudo-first-order model and b) pseudo-second-order model.

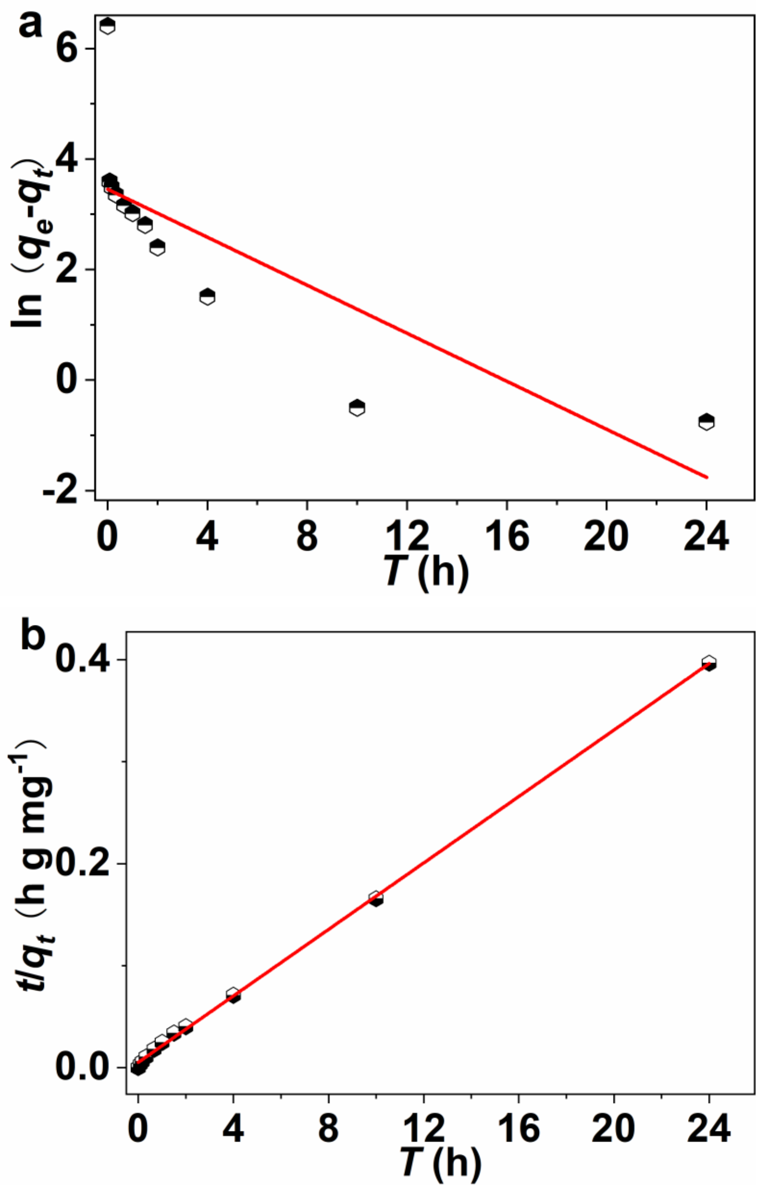

Supplementary Fig. 46 The kinetic model of OHMMCOF-1-3.0 eq as the template for Th(IV): a) pseudo-first-order model and b) pseudo-second-order model.

Supplementary Table 13. Parameters for kinetic models of U(VI)/Th(IV) adsorption by OHMMCOF-1-3.0 eq.

Nuclide	$Q_e^{(exp)}$ (mg/g)	Pseudo-first-order			Pseudo-second-order		
		k_1 (1/h)	$Q_e^{(cal)}$ (mg/g)	R_1^2	k_2 (g/mg/h)	$Q_e^{(cal)}$ (mg/g)	R_2^2
U(VI)	30.7	0.14330	4.6	0.20578	0.74150	30.7	0.99999
Th(IV)	60.5	0.21738	31.7	0.58749	0.05210	61.4	0.99934

Supplementary Section 33. XPS Spectra of OHMMCOF-2-U(VI)/Th(IV)

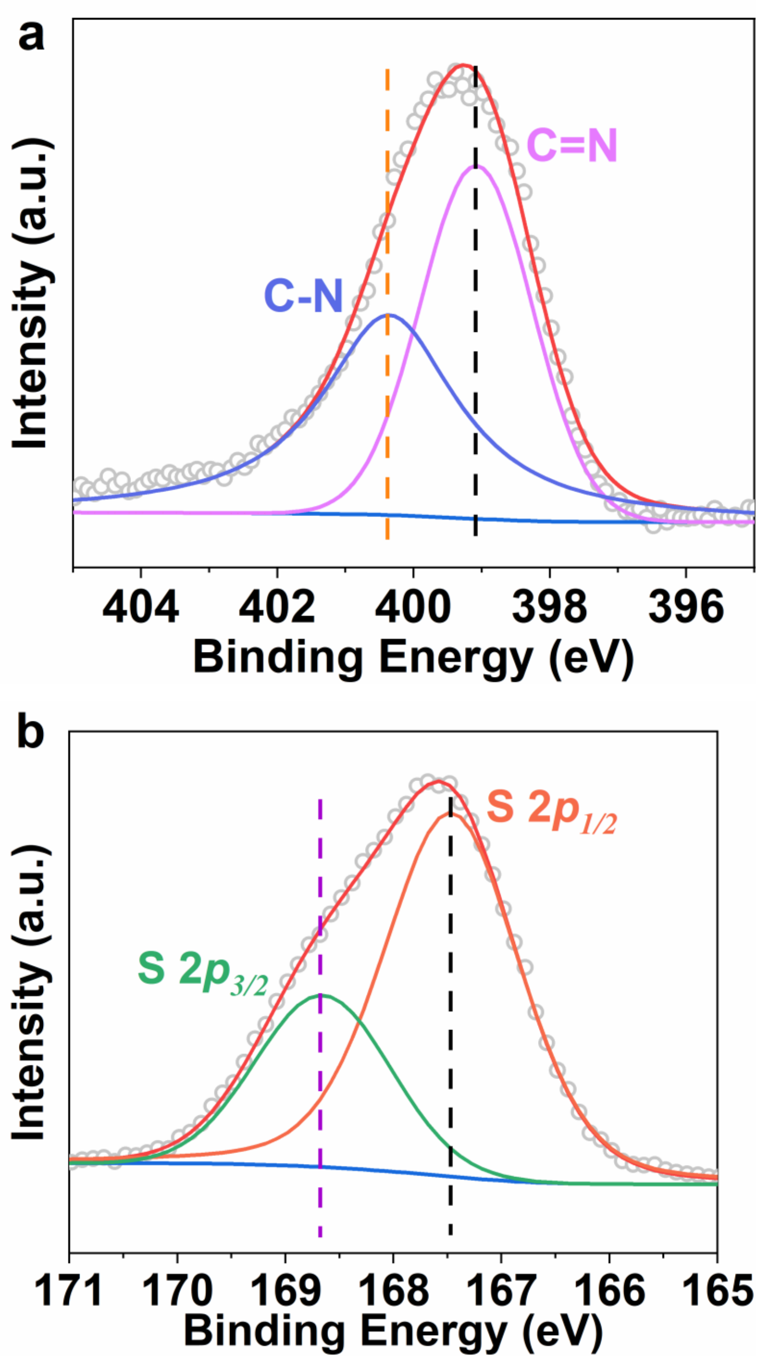

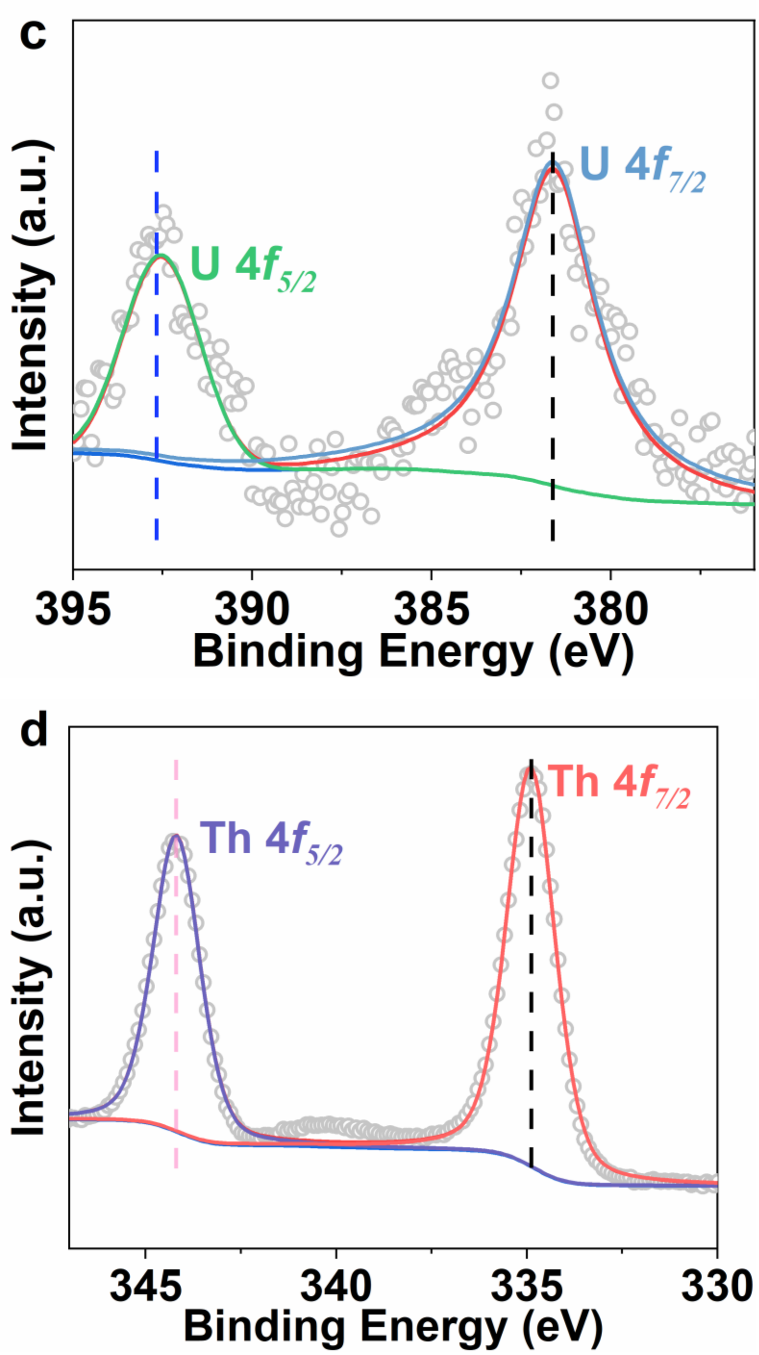

Supplementary Fig. 47 High-resolution XPS spectra of a) N 1*s*, b) S 2*p*, c) U 4*f*, and d) Th 4*f* of OHMMCOF-2-U(VI)/Th(IV).

Supplementary Section 34. SEM and TEM Images and EDS Mappings of OHMMCOF-2-U(VI)/Th(IV)

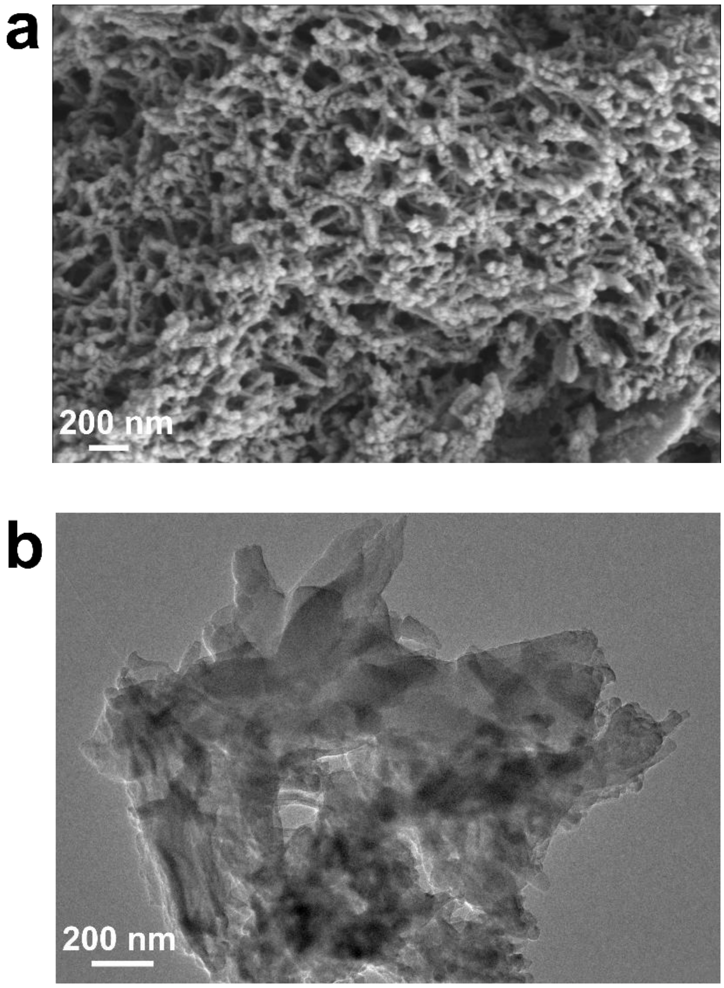

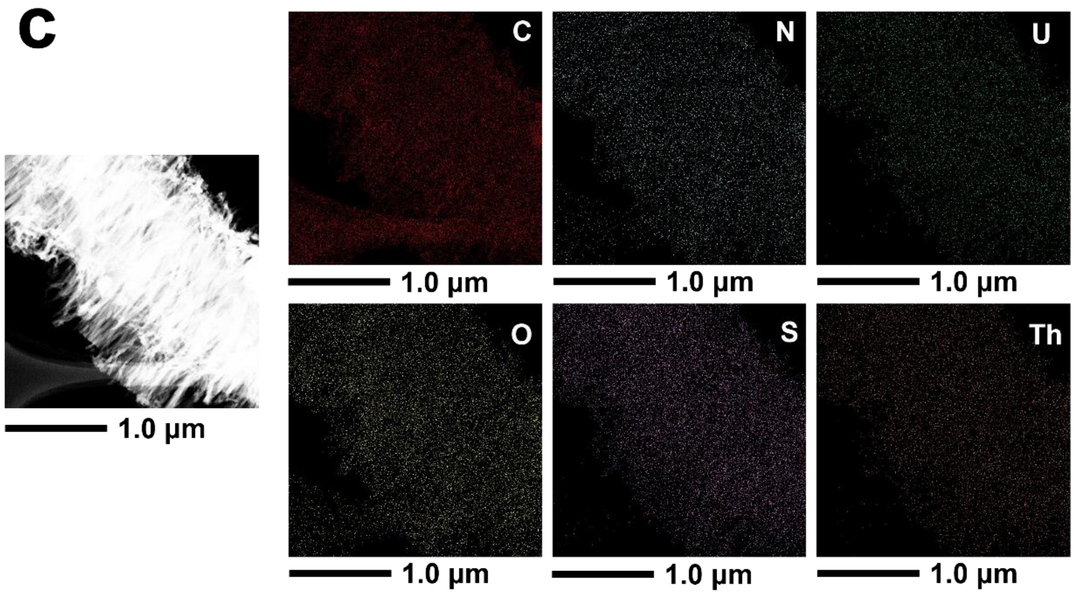

Supplementary Fig. 48 a) SEM and b) TEM images, and c) EDS mappings of OHMMCOF-2-U(VI)/Th(IV).

Supplementary Section 35. Selective Adsorption Experiment of OHMMCOF-2

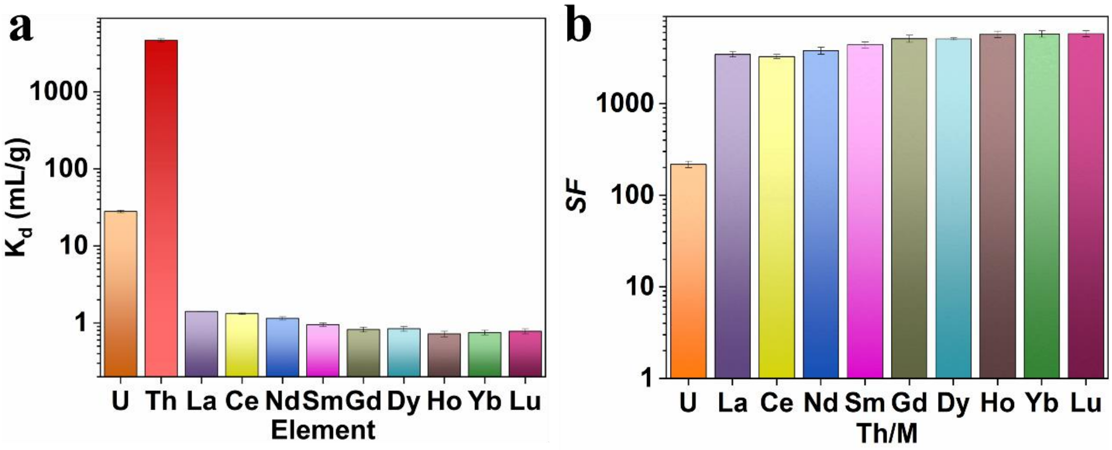

Supplementary Fig. 49 a) Distribution coefficients (K_d) and b) separation factors (*SF*) for thorium and other metals on OHMMCOF-2. All the error bars represent the standard deviation of the experiments.

Supplementary Section 36. Comparison of Th(IV) Selective Adsorption Properties of OHMMCOF-2 and Other Porous Materials

Supplementary Table 14. Comparison of Th(IV) selective adsorption properties of OHMMCOF-2 and other porous materials*.

Porous materials	Conditions	Capacity (mg/g)	K_d(Th)	*SF* (Th/U)	Ref.
GO	pH = 3.0, $C_0 \approx 23$ ppm	74.2	N.A.	36.3	6
Salen-containing MOF	pH = N.A., $C_0 = 87$ ppm	46.4	N.A.	N.A.	7
PPAC	pH = 4.0, $C_0 \approx 10$ ppm	2.8	0.33	2.1	8
WMC-O	pH = 1.7, $C_0 = 10$ ppm	8.4	1.3×10^5	~ 200	9
M-DGA	pH = 3.0, $C_0 = 90$ ppm	55.6	N.A.	N.A.	10
SBA-15-O-DMAP	pH = 1.0, $C_0 = 100$ ppm	37.5	2.5×10^3	50	11
KMS-5	pH = 1.0, $C_0 = 50$ ppm	50.0	1.04×10^6	N.A.	12
PEI-GO-AG	pH = 3.0, $C_0 = 50$ ppm	12.0	N.A.	N.A.	13
HPSM-PGAH	pH = 3.0, $C_0 = 20$ ppm	52.4	2.0×10^4	140	14
Py-TFImI-25	pH = 4.0, $C_0 = 25$ ppm	80	5.5×10^3	~ 200	15
Py-TFIm-25	pH = 4.0, $C_0 = 25$ ppm	140	1.2×10^4	~ 400	15
**OHMMCOF-2**	**pH = 3.0, $C_0 = 50$ ppm**	**71.3**	**4.6×10^3**	**218**	**This work**

* Since many literatures do not give specific adsorption rate of the first 5 min, a comprehensive comparison could not be made in the table.

Supplementary Section 37. Fractional Atomic Coordinates for the Unit Cell

Supplementary Table 15. Fractional atomic coordinates for the unit cell of MPCOF.

Space group: *P*m			
a = 22.51 Å, b = 3.48 Å, and c = 22.73 Å			
$\alpha = \gamma = 90°$ and $\beta = 119.25°$			
Rwp = 1.37 %, R_p = 1.78%			
O1	-0.47101	0	3.39312
O2	-0.57081	0	3.35603
O3	-0.58627	0	3.42991
S4	-0.51254	0	3.43421
C5	-0.92926	0	3.66748
C6	-0.85699	0	3.70115
C7	-0.82491	0	3.65982
C8	-0.86539	0	3.58948
C9	-0.93591	0	3.55837
C10	-0.96846	0	3.59725
N11	-1.04135	0	3.56705
N12	-0.46764	0	2.96538
C13	-0.29935	0	3.1309
C14	-0.3442	0	3.0612
C15	-0.41701	0	3.03569
C16	-0.44481	0	3.07955
C17	-0.40567	0	3.14944
C18	-0.33007	0	3.17977
O19	-0.16557	0	3.36315
N20	-0.28859	0	3.25713
C21	-0.31356	0	3.29775
C22	-0.08187	0	3.50243
C23	-0.18772	0	3.51275
C24	-0.15652	0	3.4724
C25	-0.19686	0	3.40195
C26	-0.2687	0	3.37168
C27	-0.29905	0	3.41285
C28	-0.25868	0	3.48338
C29	-0.28819	0	3.52848
N30	-0.3532	0	3.50692
C31	-0.36541	0	3.66557
C32	-0.33857	0	3.62198
C33	-0.38161	0	3.55149
C34	-0.4531	0	3.52521
C35	-0.4793	0	3.57028

C36	-0.43556	0	3.64039
N37	-0.46018	0	3.6878
N38	-0.75305	0	3.68285
O39	-0.66102	0	3.64096
C40	-0.52374	0	3.67233
C41	-0.70307	0	3.74367
C42	-0.45667	0	3.91484
C43	-0.51164	0	3.84436
C44	-0.49351	0	3.79375
C45	-0.54342	0	3.72545
C46	-0.61252	0	3.7078
C47	-0.63144	0	3.75873
C48	-0.58067	0	3.82672
S49	-0.81069	0	3.79501
O50	-0.87652	0	3.80562
O51	-0.72939	0	3.84394
O52	-0.80564	0	3.86826
S53	-0.30107	0	3.0094
O54	-0.33334	0	2.9286
O55	-0.23279	0	3.00303
O56	-0.22438	0	3.07242

Supplementary Table 16. Fractional atomic coordinates for the unit cell of OHMMCOF-3.

Space group: *P*-6			
a = b = 8.75 Å, and c = 3.40 Å			
α = *β* = 90°, and *γ* = 120°			
Rwp = 3.09%, Rp = 2.09%			
C1	0.31605	0.81355	0
C2	0.17558	0.65799	0
C3	0.66011	0.71392	0
O4	0.61856	0.95923	0
N5	0.64322	0.56055	0
N6	0.74754	0.49698	0
C7	0.18645	0.5025	0
C8	0.34201	0.51759	0
C9	0.28608	0.94619	0
O10	0.04077	0.65933	0
N11	0.43945	0.08267	0
N12	0.50302	0.25056	0
C13	0.4975	0.68395	0
C14	0.48241	0.82442	0
C15	0.05381	0.33989	0
O16	0.34067	0.38144	0
N17	0.91733	0.35678	0
N18	0.74944	0.25246	0
C19	0.66667	0.33333	0

Supplementary References

Supplementary Section 38. Supplementary References

1 Mitra, S. *et al.* Self-exfoliated guanidinium-based ionic covalent organic nanosheets (iCONs). *J. Am. Chem. Soc.* **138**, 2823-2828 (2016).

2 Peng, L. *et al.* One-dimensionally oriented self-assembly of ordered mesoporous nanofibers featuring tailorable mesophases via kinetic control. *Nat. Commun.* **14**, 8148 (2023).

3 Sun, H. *et al.* Lateral growth of cylinders. *Nat. Commun.* **13**, 2170 (2022).

4 Ren, Y. *et al.* Dynamic coassembly of amphiphilic block copolymer and polyoxometalates in dual solvent systems: an efficient approach to heteroatom-doped semiconductor metal oxides with controllable nanostructures. *ACS Cent. Sci.* **8**, 1196-1208 (2022).

5 Uribe-Romo, F. J. *et al.* A crystalline imine-linked 3-D porous covalent organic framework. *J. Am. Chem. Soc.* **131**, 4570-4571 (2009).

6 Jiang, D. *et al.* The separation of Th(IV)/U(VI) via selective complexation with graphene oxide. *Chem. Eng. J.* **271**, 147-154 (2015).

7 Guo, X. G., Qiu, S., Chen, X., Gong, Y. & Sun, X. Postsynthesis modification of a metallosalen-containing metal-organic framework for selective Th(IV)/Ln(III) separation. *Inorg. Chem.* **56**, 12357-12361 (2017).

8 Xiong, J. *et al.* Polypropylene modified with amidoxime/carboxyl groups in separating uranium(VI) from thorium(IV) in aqueous solutions. *ACS Sustainable Chem. Eng.* **5**, 1924-1930 (2017).

9 Wang, Z., Brown, A. T., Tan, K., Chabal, Y. J. & Balkus, K. J., Jr. Selective extraction of thorium from rare earth elements using wrinkled mesoporous carbon. *J. Am. Chem. Soc.* **140**, 14735-14739 (2018).

10 Hu, Y. *et al.* Selective separation and preconcentration of Th(IV) using organo-functionalized, hierarchically porous silica monoliths. *J. Mater. Chem. A* **7**, 289-302 (2019).

11 Zhang, F. *et al.* Selective separation of thorium from rare earths and uranium in acidic solutions by phosphorodiamidate-functionalized silica. *Chem. Eng. J.* **392**, 123717 (2020).

12 Xu, L. *et al.* Selective capture mechanism of radioactive thorium from highly acidic solution by a layered metal sulfide. *ACS Appl. Mater. Interfaces* **13**, 37308-37315 (2021).

13 Bai, R. *et al.* Polyethylenimine functionalized and scaffolded graphene aerogel and the application in the highly selective separation of thorium from rare earth. *Mater. Design* **197**, 109195 (2021).

14 Ma, K.-Q. *et al.* Advanced solid-phase extraction of tetravalent actinides using a novel hierarchically porous functionalized silica monolith. *Sep. Purif. Technol.* **293**, 121086 (2022).

15 Liu, X. *et al.* Efficient and selective capture of thorium ions by a covalent organic framework. *Nat. Commun.* **14**, 5097 (2023).
